# Physiological and transcriptome analyses for assessing the effects of exogenous uniconazole on drought tolerance in hemp (*Cannabis sativa* L.)

**DOI:** 10.1038/s41598-021-93820-6

**Published:** 2021-07-14

**Authors:** Ying Jiang, Yufeng Sun, Dianfeng Zheng, Chengwei Han, Kun Cao, Lei Xu, Shuxia Liu, Yanyong Cao, Naijie Feng

**Affiliations:** 1 College of Agronomy, Heilongjiang Bayi Agricultural University, Daqing, 163316 China; 2 Daqing Branch of Heilongjiang Academy of Sciences, Daqing, 163319 China; 3 College of Coastal Agricultural Sciences, Guangdong Ocean University, Zhanjiang, 524088 China; 4 Shenzhen Research Institute of Guangdong Ocean University, Shenzhen, 518108 China; 5 Institute of Cereal Crops, Henan Academy of Agricultural Sciences, Zhengzhou, 450002 China

**Keywords:** Plant genetics, Plant physiology, Plant stress responses

## Abstract

Uniconazole (S-(+)-uniconazole), a plant growth retardant, exerts key roles in modulating growth and development and increasing abiotic stress tolerance in plants. However, the underlying mechanisms by which uniconazole regulates drought response remain largely unknown. Here, the effects of exogenous uniconazole on drought tolerance in hemp were studied via physiological and transcriptome analyses of the drought-sensitive industrial hemp cultivar Hanma No. 2 grown under drought stress. Exogenous uniconazole treatment increased hemp tolerance to drought-induced damage by enhancing chlorophyll content and photosynthesis capacity, regulating activities of enzymes involved in carbon and nitrogen metabolism, and altering endogenous hormone levels. Expression of genes associated with porphyrin and chlorophyll metabolism, photosynthesis-antenna proteins, photosynthesis, starch and sucrose metabolism, nitrogen metabolism, and plant hormone signal transduction were significantly regulated by uniconazole compared with that by control (distilled water) under drought stress. Numerous genes were differentially expressed to increase chlorophyll content, enhance photosynthesis, regulate carbon–nitrogen metabolism-related enzyme activities, and alter endogenous hormone levels. Thus, uniconazole regulated physiological and molecular characteristics of photosynthesis, carbon–nitrogen metabolism, and plant hormone signal transduction to enhance drought resistance in industrial hemp.

## Introduction

Drought stress, an abiotic stress, has more important effects on crop yield and quality compared to other abiotic stresses, inducing a highly vulnerable state in plants because of high temperature and low water content^1^. The photosynthetic system is greatly damaged owing to drought-induced low leaf water content, membrane lipid peroxidation, damaged leaf microstructure, higher leaf cell membrane permeability, and decreased photosynthetic pigment content^2^. Photosynthesis, as an index of the photosynthetic system, regulates key processes in carbon metabolism. Drought stress reduces net photosynthetic rate and carbon fixation capacity, which affects carbon absorption and metabolism in plants^3^. Therefore, carbon metabolism must be tightly regulated to enhance drought resistance in plants by changing starch content, with extensive accumulation of soluble sugar and sucrose^4^, and inverting sucrose catalyzed by sucrose synthase (SS) and sucrose phosphate synthase (SPS)^5^. Nitrogen metabolism is closely related to photosynthesis and carbon metabolism and is significant for plants adapting to environmental changes. The activities of key enzymes involved in N metabolism may play a major role in plant photosynthetic adaptation under drought stress^6^. Tolerance to drought stress is also manifested as increased levels of drought-responsive proteins and the expression of genes involved in signal transduction^7^. Plants initiate a series of morphological, molecular, and physiological and biochemical changes such as hormone regulation and gene expression to tolerate drought stress^8^. It is thus vital to study the mechanisms underlying drought stress resistance to develop effective strategies for alleviating drought-induced damage and improving agricultural production.

Hemp (*Cannabis sativa* L.) has one of the longest histories among fibre and traditional economic crops. It provides major raw materials such as fibre, seeds, xyloid stems, and floral leaves for numerous traditional and innovative industries globally^9–11^. Industrial hemp has a delta-9-tetrahydrocannabinol content of ≤ 0.3%, according to international standards. Drought stress during the growth period of hemp can lead to an increased incidence of diseases and insect pests, slow growth, and delay fibre and seed maturation, which ultimately affects yield and quality^11^. Few studies have focused on the mechanisms underlying drought stress resistance in hemp^12^. Currently, physiological and transcriptome analyses are widely used in plant science to assess changes that occur under drought stress but have been rarely utilized to study such changes in hemp.

Plant growth regulators can be used to regulate plant growth and stress tolerance under conditions of abiotic stress. Uniconazole, as one of the most efficient plant growth regulators, alters different parameters involved in the growth and development of plants under environmental stress^13^. Uniconazole applied exogenously under stress increases resistance to freezing injury^14^, water deficit stress^15^, drought stress^16^, waterlogging-induced damage^17^, and salt stress^18^. Previous studies^14–18^ revealed that uniconazole improved net photosynthesis and transpiration rate, suggesting an increase in leaf water potential and chlorophyll (Chl) content. Uniconazole also has various functions in promoting proline and soluble sugar accumulation, strengthening the antioxidant defence system, stimulating protein levels and nitrate reductase (NR) activity, and altering endogenous hormone levels. These experiments were conducted for different plants, such as soybean, bean, winter rape, and datura. However, the response of hemp to uniconazole is unclear. Research in other plants has revealed that uniconazole produces a marked effect on plant hormone metabolism. It induced a reduction in the content of gibberellin-like substances, mainly by inhibiting the cytochrome P450 enzyme *ent-kaurene* oxidase, which catalyses the three oxidation steps of transformation from *ent-kaurene* to *ent-kaurenoic* acid^19^. Uniconazole also suppressed a major abscisic acid (ABA) catabolic enzyme, ABA 8’-hydroxylase^20^. In addition, Liu et al.^21,22^ performed transcriptome analysis to dissect uniconazole-regulated expression of genes involved in the starch metabolism pathway. Furthermore, transcriptome analysis was applied to identify the complex molecular mechanisms underlying root development after treatment with uniconazole^23^. The effects of uniconazole on key enzymes involved in plant hormone signal transduction, carbohydrate metabolism, and starch metabolism pathways had been investigated. However, the genes and metabolic pathways involved in the response of hemp to uniconazole treatment under drought stress are unknown.

In the present study, we investigated the influences of uniconazole on the physiological and genome-wide gene expression of *Cannabis sativa* L. under drought stress. Our findings potentially lead to a thorough understanding of the biological function and the molecular mechanisms underlying uniconazole activity in hemp under drought stress.

## Results

### Effect of uniconazole on Chl content and net photosynthetic rate under drought stress

**Figure 1:**
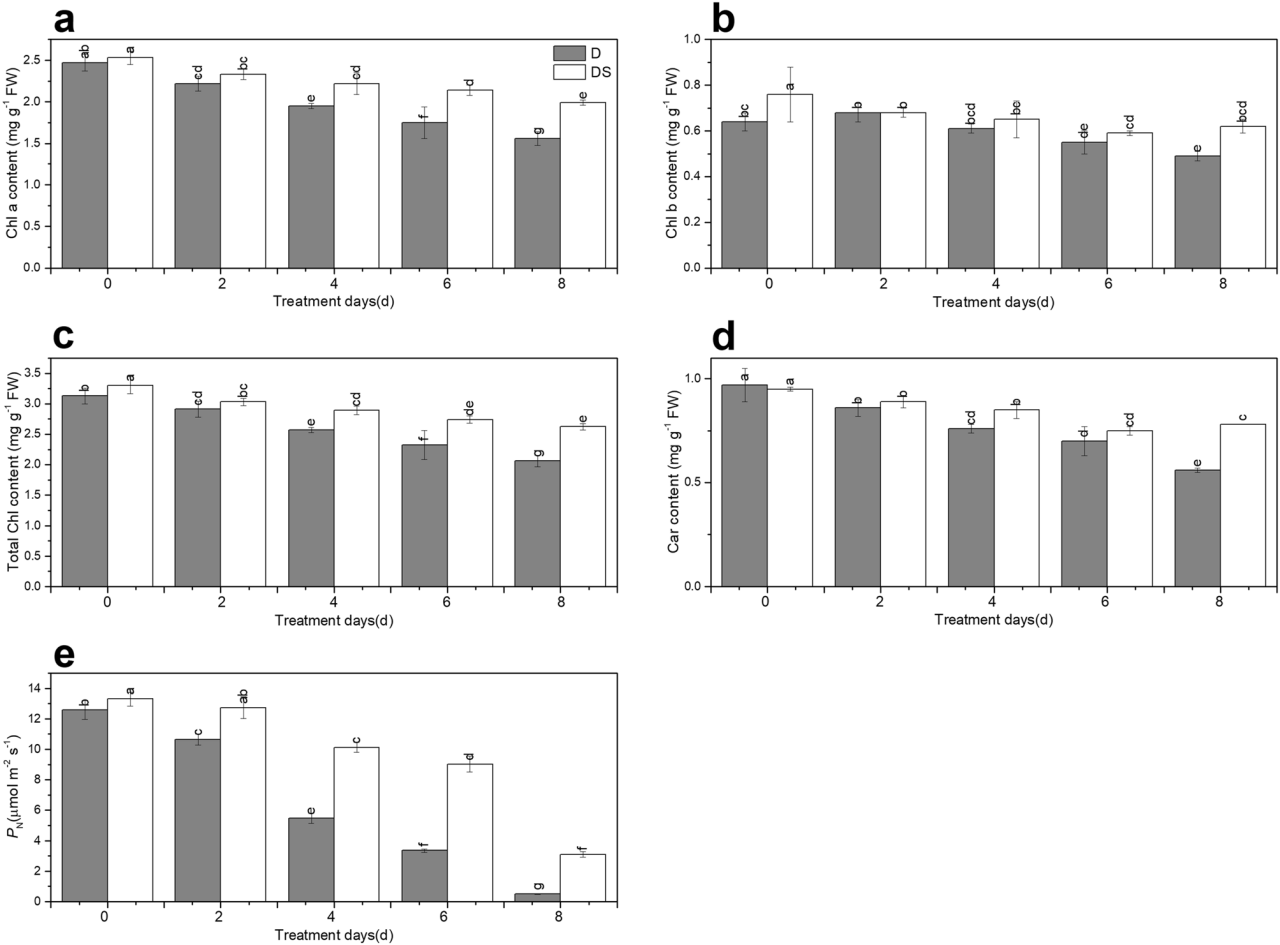
Uniconazole-induced chlorophyll content (**a**–**d**) and net photosynthetic rate (**e**) under drought stress. All data are presented as the means ± standard errors (n = 4). D and DS expressed as treatments under drought stress and drought stress + uniconazole, respectively.

Drought stress decreased Chl content, but uniconazole mitigated this downward trend prominently (Fig. 1). Chl a content in plants treated with uniconazole (DS) was 13.9%, 22.3%, and 27.6% higher than that in drought-treated (D) plants following exposure to drought stress for 4, 6, and 8 days, respectively (Fig. 1a). There was a significant increase in Chl b content of 26.5% in DS plants compared to D plants under drought stress for 8 days (Fig. 1b). The total Chl content of DS plants was 4.1–27.2% higher than that of D plants (Fig. 1c). At 4 and 8 days, carotenoid (Car) content rapidly improved by 11.8% and 39.3% in DS plants, respectively, compared with that in D plants (Fig. 1d).

Net photosynthetic rate (*P*_N_) was reduced in the leaves of industrial hemp subjected prolonged drought stress (Fig. 1e). Uniconazole treatment sharply increased *P*_N_ of plants by 1.1–6.0-fold relative to D plants under drought stress (Fig. 1e).

### Effect of uniconazole on carbon metabolism-related enzyme activities under drought stress

**Figure 2:**
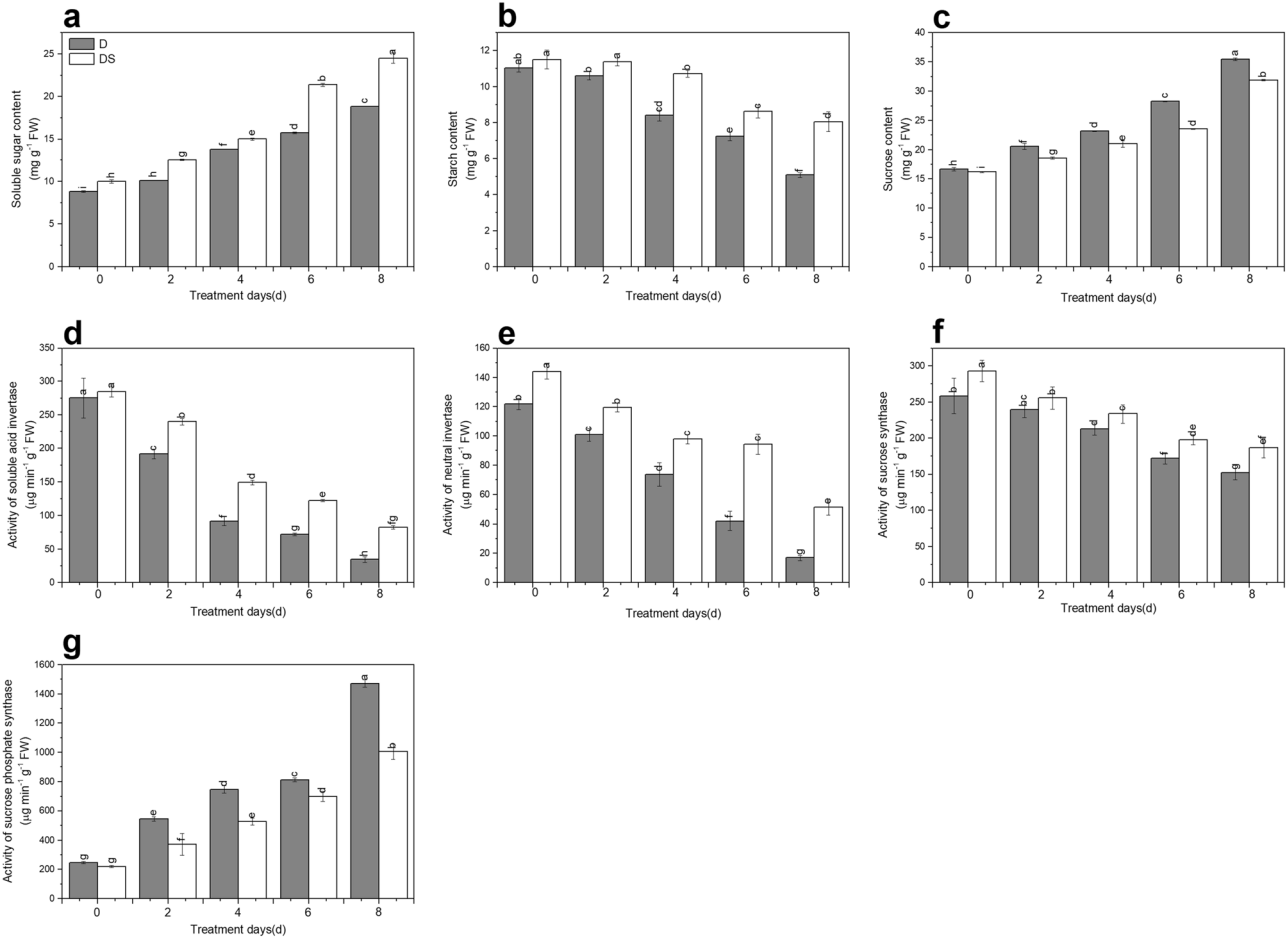
Uniconazole-induced carbon metabolism-related indicators as soluble sugar content (**a**), starch content (**b**), sucrose content (**c**), activity of soluble acid invertase (**d**), activity of neutral invertase (**e**), activity of sucrose synthase (**f**) and activity of sucrose phosphate synthase (**g**) under drought stress. All data are presented as the means ± standard errors (n = 4). D and DS expressed as treatments under drought stress and drought stress + uniconazole, respectively.

Soluble sugar content sharply increased under prolonged drought stress. DS plants showed significant increases of 9.0–30.0% in soluble sugar content relative to D plants (Fig. 2a). Drought caused a drastic decrease in starch content. While exogenous uniconazole treatment alleviated this decrease in starch content that was 7.3%, 27.5%, 19.2%, and 58.2% higher than that in D plants following exposure to drought stress for 2, 4, 6, and 8 days, respectively (Fig. 2b). There was a dramatic increase in sucrose content with the prolonged drought stress. However, DS plants showed reduced sucrose content by 2.7–16.9% compared with D plants (Fig. 2c). Soluble acid invertase (S-AI), neutral invertase (NI), and SS activities reduced with the extension of drought stress. Exogenous uniconazole application relieved this reduction in S-AI, NI and SS activities compared with no application under drought stress (Fig. 2d–f). The SPS activity increased following drought stress for 2, 4, 6, and 8 days, which was decreased significantly by 31.9%, 29.4%, 13.8%, and 31.7% in DS plants, respectively, compared with that in D plants (Fig. 2g).

### Effect of uniconazole on nitrogen metabolism-related enzyme activities under drought stress

**Figure 3:**
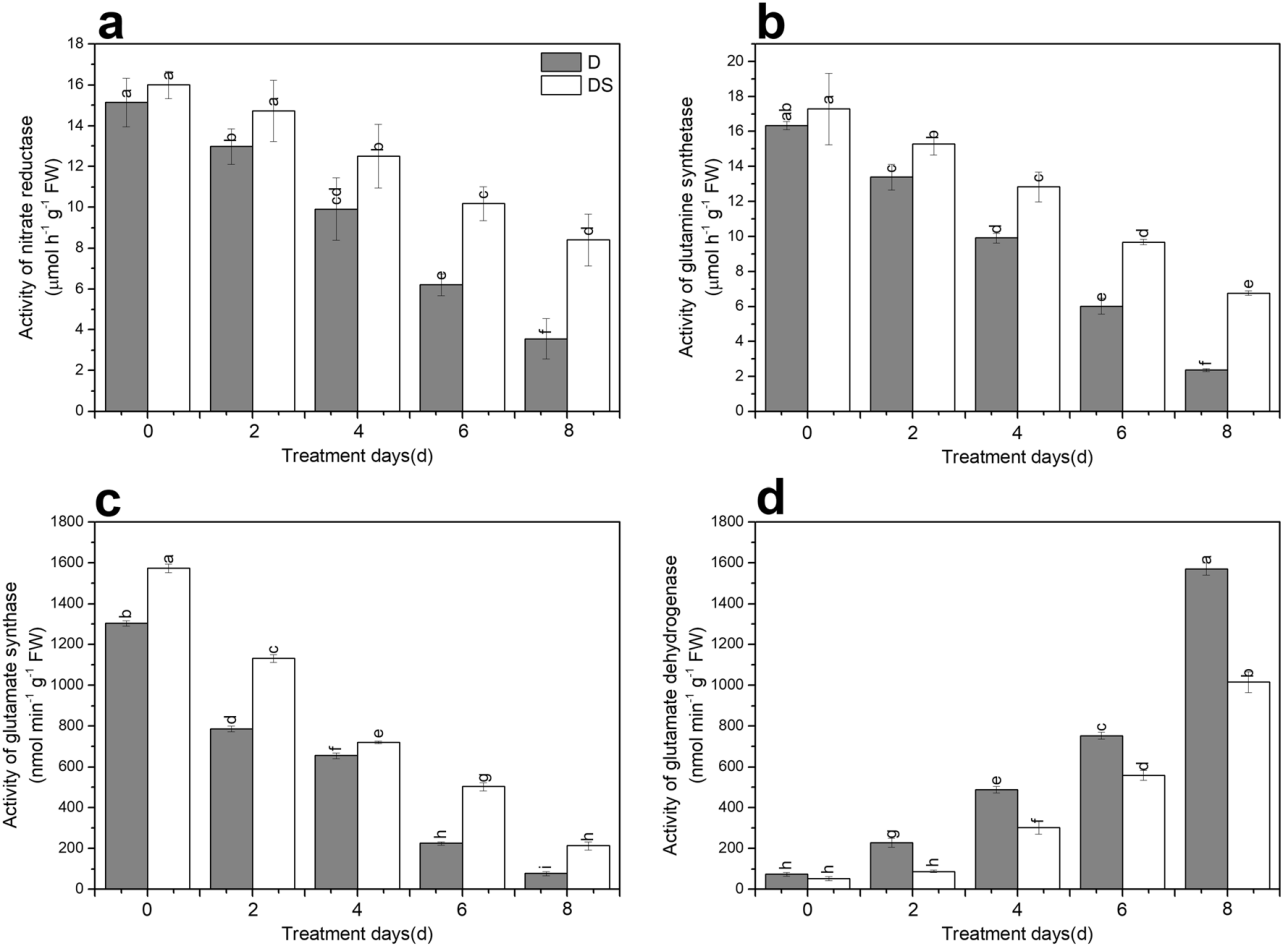
Uniconazole-induced nitrogen metabolism-related indicators as activity of nitrate reductase (**a**), activity of glutamine synthetase (**b**), activity of glutamate synthase (**c**) and activity of glutamate dehydrogenase (**d**) under drought stress. All data are presented as the means ± standard errors (n = 4). D and DS expressed as treatments under drought stress and drought stress + uniconazole, respectively.

NR activity, glutamine synthetase (GS) activity and glutamate synthase (GOGAT) activity in the leaves of seedlings decreased under drought stress. Application of uniconazole to seedlings under drought stress for 2, 4, 6, and 8 days increased NR activity sharply by 13.5%, 26.2%, 64.4%, and 136.0%, respectively, compared to that non-treated seedlings (Fig. 3a). Similarly, under drought stress for 2, 4, 6, and 8 days, DS plants showed significantly improved GS activity by 1.1-fold, 1.3-fold, 1.6-fold, and 2.9-fold, respectively, compared with D plants (Fig. 3b). Additionally, DS plants dramatically increased GOGAT activity (1.1–2.8-fold) compared to D plants (Fig. 3c). The activity of glutamate dehydrogenase (GDH) increased with the aggravation of drought stress. Exogenous uniconazole supplementation reversed this trend of increased GDH activity that was a decrease of 61.3%, 38.2%, 25.9%, and 35.3%, respectively, in DS plants compared with that in D plants under drought stress for 2, 4, 6, and 8 days (Fig. 3d).

### Effect of uniconazole on endogenous hormone levels under drought stress

**Figure 4:**
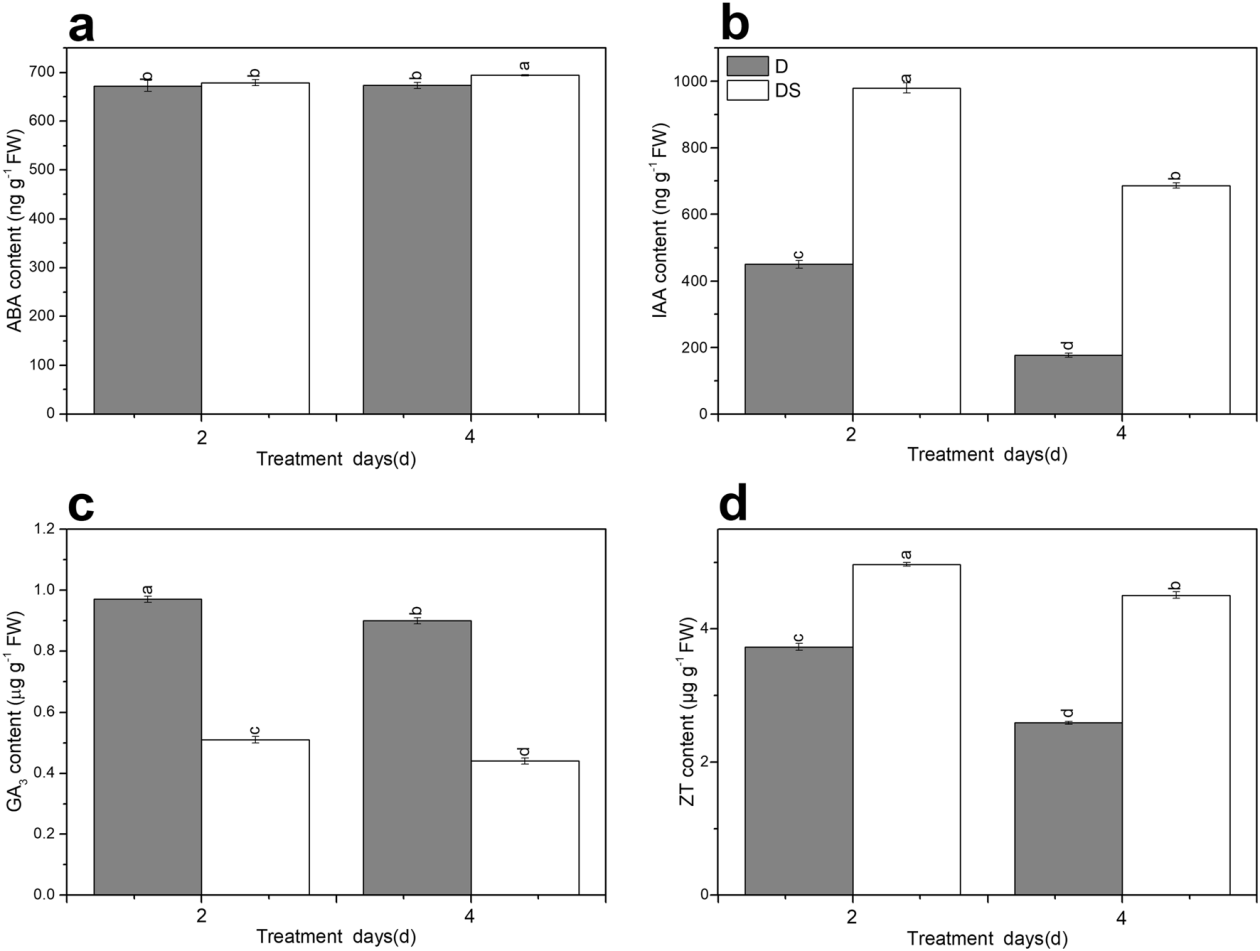
Uniconazole-induced endogenous hormones of ABA (**a**), IAA (**b**), GA_3_ (**c**), ZT (**d**) levels under drought stress. All data are presented as the means ± standard errors (n = 3). D and DS expressed as treatments under drought stress and drought stress + uniconazole, respectively.

ABA content increased with the prolonged drought stress, but the difference was not significant (Fig. 4a). Under drought stress for 4 days, ABA content of DS plants apparently increased by 2.2% compared with that of D plants (Fig. 4a). The content of indole-3-acetic acid (IAA), gibberellic acid (GA_3_), and zeatin (ZT) markedly decreased by 60.7%, 7.2%, and 30.6%, respectively, under drought stress for 4 days compared with those in plants under drought stress for 2 days (Fig. 4b–d). Compared with untreated plants, exogenous uniconazole application apparently increased IAA content by 2.2-fold and 3.9-fold in plants under drought stress for 2 and 4 days, respectively (Fig. 4b). GA_3_ content in DS plants was 47.4% and 51.1% lower than that in D plants under drought stress for 2 and 4 days, respectively (Fig. 4c). Compared to plants without supplementation, uniconazole significantly enhanced ZT content by 33.2% and 74.1% under drought stress for 2 and 4 days, respectively (Fig. 4d).

### Effect of uniconazole on the expression of differentially expressed genes (DEGs) under drought stress

Twelve samples were collected for transcriptome sequencing, the results from Supplementary Table S1 revealed that the selected reference genome assembly could meet the needs of information analysis. Identification of DEGs revealed 5736 DEGs between D2_vs_DS2 and D4_vs_DS4 (drought stress for 2 and 4 days/drought stress treated with uniconazole for 2 and 4 days). Among them, 1701 DEGs were commonly regulated across different treatment periods, accounting for 29.65%. Non-shared DEGs were 1071 and 2964 in number, accounting for 18.67% and 51.67%, respectively (Supplementary Fig. S1a). The results indicated that the exogenous application of uniconazole had already mobilized few genes to counteract the damage caused by drought stress for 2 days and promoted strong drought resistance ability after 4 days of drought stress.

The expression trends of 21 genes were identical between the results of RNA-Seq and qRT-PCR. Thus, the RNA-Seq data was reliable (Supplementary Fig. S1b, Supplementary Table S10).

**Figure 5:**
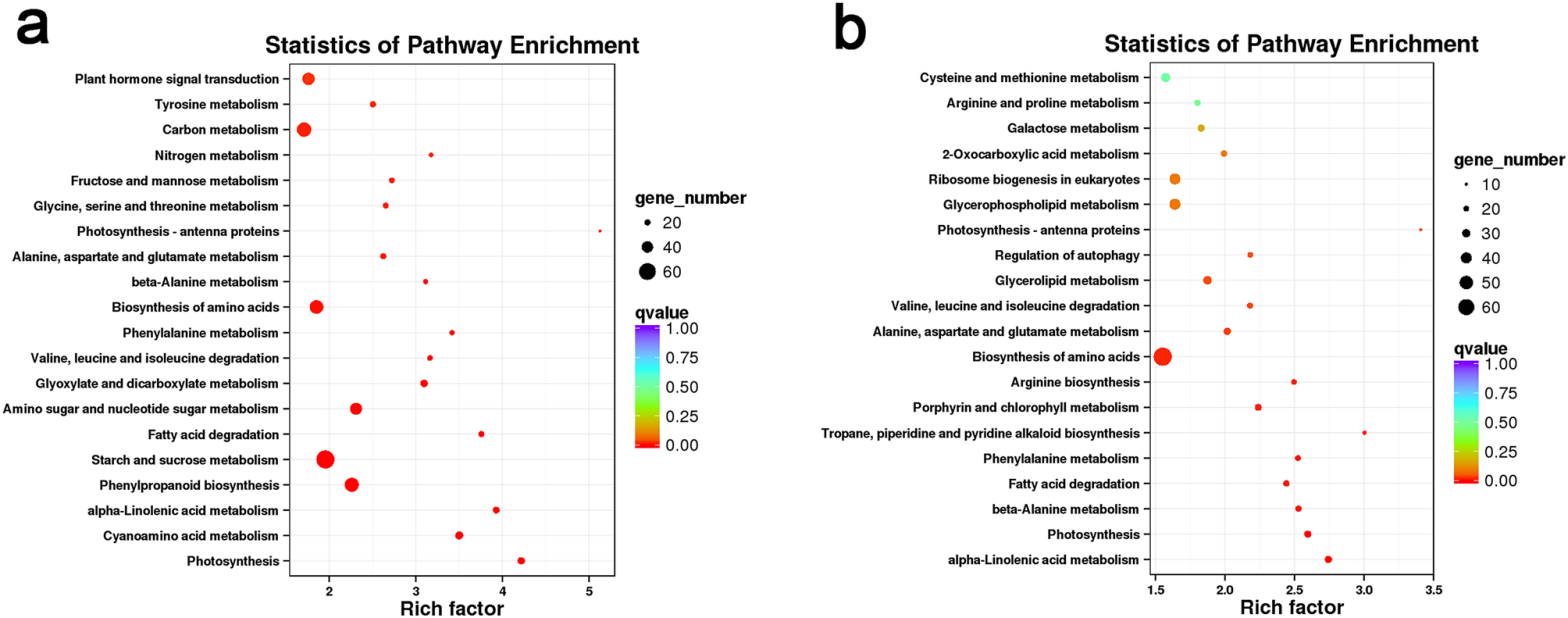
KEGG enrichment analysis^83–85^ of the DEGs between D and DS. **a**, **b**, KEGG enrichment analysis of differentially expressed genes of D_vs_DS for 2 days and 4 days, respectively. Each circle represented a KEGG pathway, the ordinate represented the name of the pathway, and the abscissa represented the enrichment factor. The circle color represented q value, the circle size represented the number of genes enriched in the pathway.

Kyoto Encyclopedia of Genes and Genomes (KEGG) enrichments at D_vs_DS was performed, with the top 20 metabolic pathways shown in Fig. 5. Pathways related to photosynthesis (KO: ko00195), starch and sucrose metabolism (KO: ko00500), photosynthesis-antenna proteins (KO: ko00196), porphyrin and Chl metabolism (KO: ko00860), nitrogen metabolism (KO: ko00910), plant hormone signal transduction (KO: ko04075), and carbon metabolism (KO: ko01200) were significantly enriched by uniconazole under drought stress.

### DEGs in porphyrin and Chl metabolism, photosynthesis-antenna proteins, and photosynthesis under drought stress

**Figure 6:**
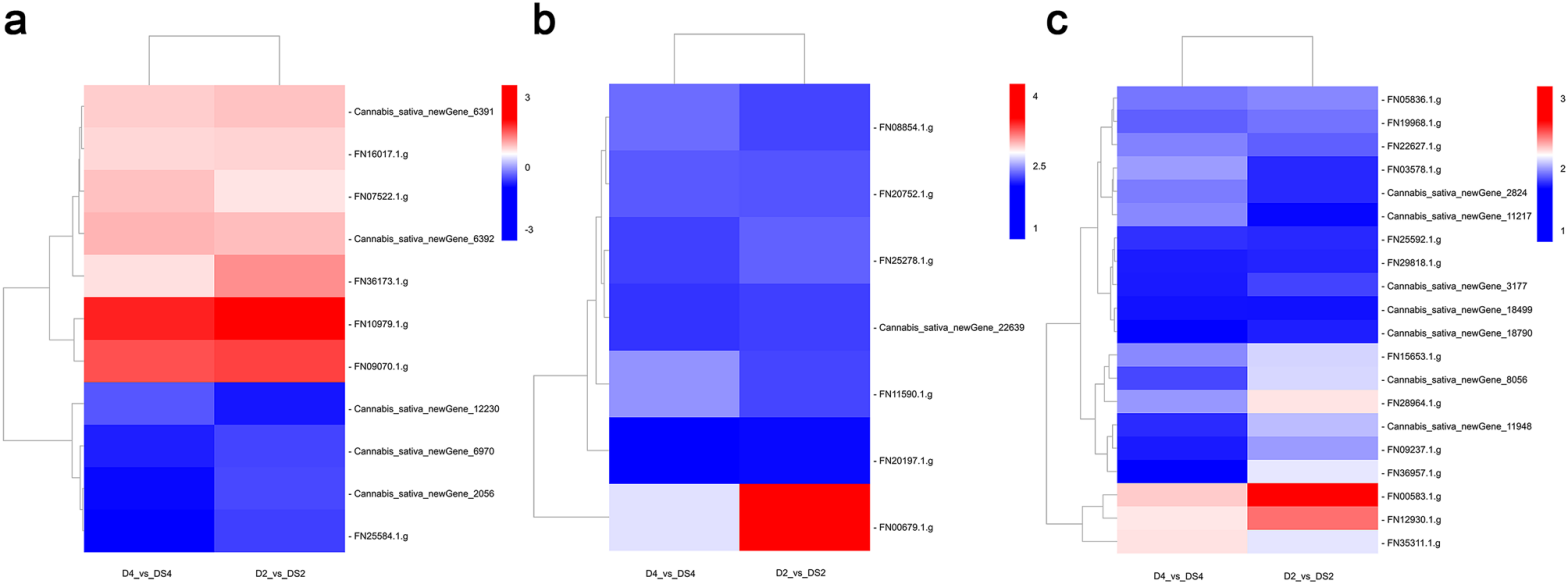
Heatmap of the DEGs in porphyrin and chlorophyll metabolism, photosynthesis-antenna proteins and photosynthesis between D and DS. The expression level changes of genes in each group were described as log_2_ fold change of FPKM. Colors indicated up- and down-regulated levels of genes. Uniconazole-induced expression patterns of 11 porphyrin and chlorophyll metabolism genes under drought stress (**a**). Uniconazole-induced expression patterns of 7 photosynthesis-antenna proteins genes under drought stress (**b**). Uniconazole-induced expression patterns of 20 photosynthesis genes under drought stress (**c**). D2_vs_DS2 means drought stress for 2 days/drought stress treated with uniconazole for 2 days; D4_vs_DS4 means drought stress for 4 days/drought stress treated with uniconazole for 4 days. The heatmap was constructed using the drawing tools of Biomarker Technologies Co., Ltd. (Beijing, China) (https://international.biocloud.net/zh/software/tools/heatmap/-1/-1).

Among 27 DEGs involved in porphyrin and Chl metabolism, 11 were regulated commonly to the two comparisons (D2_vs_DS2 and D4_vs_DS4) (Fig. 6a). In the present study, 7 porphyrin and Chl metabolism related genes were up-regulated and 4 related genes were down-regulated at D2_vs_DS2 and D4_vs_DS4 (Supplementary Table S2). Glutamyl-tRNA reductase gene (*hemA*; FN36173.1.g) was significantly up-regulated by uniconazole under drought stress. Uniconazole induced up-regulation of genes involved in Chl biosynthesis, such as magnesium (Mg) chelatase subunit gene (*chlH*; FN16017.1.g), Mg protoporphyrin IX monomethyl ester (oxidative) cyclase genes (*acsF*, *chlE*; Cannabis_sativa_newGene_6391, and Cannabis_sativa_newGene_6392), and protochlorophyllide reductase gene (*por*; FN07522.1.g) and increased the intermediate products in the synthetic pathway and contents of Chl a. However, it down-regulated chlorophyll(ide) b reductase genes (*NOL*, *NYC1*; Cannabis_sativa_newGene_2056, FN25584.1.g, and Cannabis_sativa_newGene_6970) in plants grown under drought stress.

Photosynthesis-antenna proteins play major roles in photosynthesis. Among 10 DEGs in photosynthesis-antenna proteins, 7 were regulated commonly to the two comparisons (Fig. 6b). All genes were Chl a/b-binding protein genes (*LHCA2*, *LHCA3*, *LHCA4*, *LHCA5*, *LHCB1*, *LHCB5*; FN08854.1.g, Cannabis_sativa_newGene_22639, FN20752.1.g, FN20197.1.g, FN00679.1.g, FN11590.1.g, and FN25278.1.g), which were up-regulated by uniconazole in plants grown under drought stress (Supplementary Table S3).

In this study, of the 29 DEGs in photosynthesis, 20 were up-regulated commonly to the two comparisons (Fig. 6c). Three photosystem II oxygen-evolving enhancer protein genes (*psbQ*, *psbP*; FN36957.1.g, FN29818.1.g, and Cannabis_sativa_newGene_3177) and two photosystem II reaction centre genes (*psb28*, *psbW*; FN28964.1.g and FN35311.1.g) exerted primary roles in photosystem II. Photosystem I reaction centre subunit genes (*psaD*, *psaG*, *psaH*, *psaK*, *psaL*, *psaE*; FN19968.1.g, FN09237.1.g, FN05836.1.g, FN12930.1.g, FN25592.1.g, Cannabis_sativa_newGene_11948, and Cannabis_sativa_newGene_8056) catalysed the transfer of electrons from PC to ferredoxin (FD) through a series of electron transporters. One cytochrome c6 gene (*petJ*; Cannabis_sativa_newGene_11217), one plastocyanin (PC) gene (*petE*; FN22627.1.g), four FD genes (*petF*; Cannabis_sativa_newGene_2824, FN00583.1.g, FN03578.1.g, and FN15653.1.g) and two FD-NADP reductase genes (*petH*; Cannabis_sativa_newGene_18499 and Cannabis_sativa_newGene_18790) were associated with photosynthetic electron transport (Supplementary Table S4).

### DEGs in starch, sucrose, and nitrogen metabolisms under drought stress

**Figure 7:**
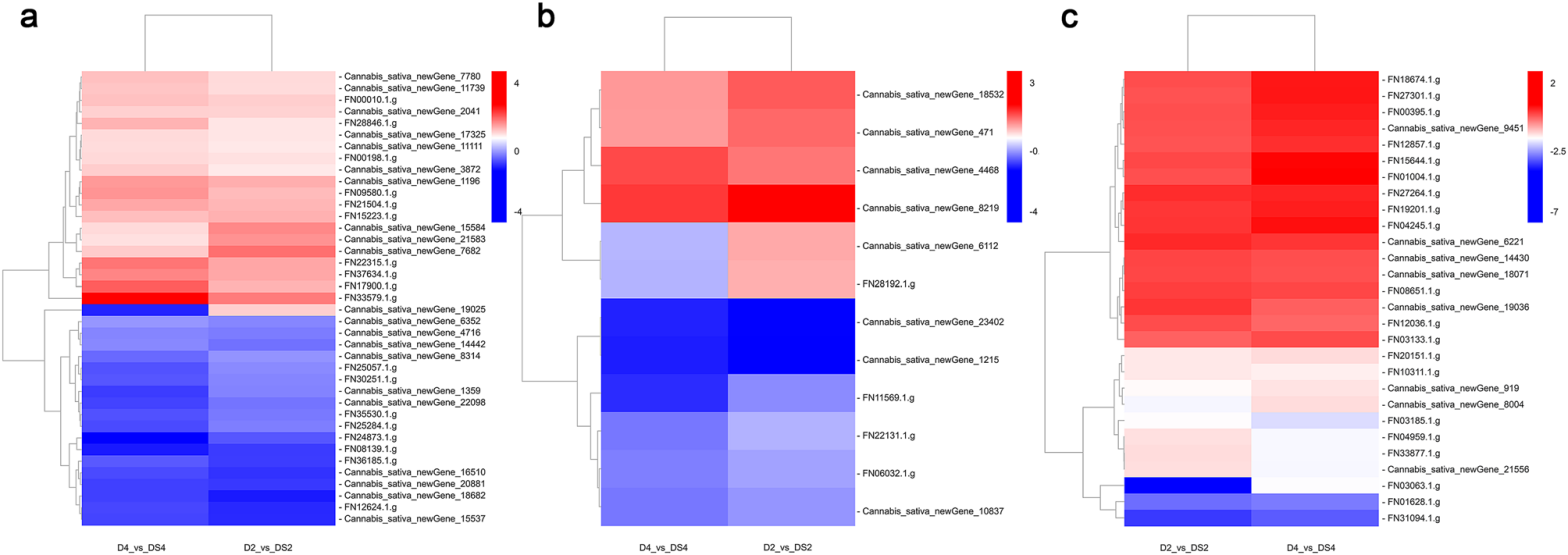
Heatmap of the DEGs in starch and sucrose metabolism, nitrogen metabolism and plant hormone signal transduction metabolism between D and DS. The expression level changes of genes in each group were described as log_2_ fold change of FPKM. Colors indicated up- and down-regulated levels of genes. Uniconazole-induced expression patterns of 39 starch and sucrose metabolism genes under drought stress (**a**). Uniconazole-induced expression patterns of 12 nitrogen metabolism genes under drought stress (**b**). Uniconazole-induced expression patterns of 28 plant hormone signal transduction metabolism genes under drought stress (**c**). D2_vs_DS2 means drought stress for 2 days/drought stress treated with uniconazole for 2 days; D4_vs_DS4 means drought stress for 4 days/drought stress treated with uniconazole for 4 days. The heatmap was drawn using the BMKCloud tools (Biomarker Technologies Co., Ltd., Beijing, China) (https://international.biocloud.net/zh/software/tools/heatmap/-1/-1).

Among 98 DEGs in the starch and sucrose metabolism pathways, five sucrose-phosphate synthase genes (*SPS*; FN15293.1.g, FN06206.1.g, FN08251.1.g, FN19565.1.g, and FN33647.1.g) were identified in sucrose metabolism. Among them, FN15293.1.g was down-regulated at D2 compared with that at DS2, but not differentially expressed at D4 relative to that at DS4. Four other genes were not differentially expressed at D2 compared with that at DS2 but were down-regulated at D4 compared with that at DS4 (Supplementary Table S5). Of the 98 DEGs identified, 39 in the starch and sucrose metabolism pathways were regulated similarly (Fig. 7a). Two sucrose synthase genes (*SuSy*; Cannabis_sativa_newGene_18682 and FN12624.1.g) were down-regulated by uniconazole in plants grown under drought stress. In addition, one beta-fructofuranosidase gene (*INV*), four fructokinase genes (*scrK*), eight beta-glucosidase genes (*bglX*), three glucan endo-1,3-beta-glucosidase genes (*GN4*), two glucose-1-phosphate adenylyltransferase genes (*glgC*), three glycogen phosphorylase genes (*PYG*), two 1,4-alpha-glucan branching enzyme genes (*GBE1*), one beta-amylase gene, seven trehalose 6-phosphate synthase/phosphatase genes (*TPS*), and three alpha-trehalase genes (*TREH*), which are involved in the synthesis and hydrolysis of starch, sucrose, fructose and glucose, were induced by uniconazole in plants grown under drought stress (Supplementary Table S6).

Plants adjust to abiotic stress by regulating key enzymes involved in nitrogen metabolism. Among 16 DEGs in the nitrogen metabolic pathways, 12 were regulated commonly to the two comparisons (Fig. 7b). One nitrate/nitrite transporter (NRT) gene (*NRT*; Cannabis_sativa_newGene_4468) was up-regulated by uniconazole in plants grown under drought stress. One glutamine synthetase gene (*glnA*; FN22131.1.g) and two glutamate synthase genes (*GLT1*; Cannabis_sativa_newGene_10837 and FN06032.1.g) were down-regulated by uniconazole in plants grown under drought stress. Among four glutamate dehydrogenase genes (*gdhA*; Cannabis_sativa_newGene_1215, Cannabis_sativa_newGene_23402, Cannabis_sativa_newGene_6112, and FN28192.1.g) of interest, Cannabis_sativa_newGene_1215 and Cannabis_sativa_newGene_23402 were down-regulated and Cannabis_sativa_newGene_6112 and FN28192.1.g were up-regulated by uniconazole in plants grown under drought stress at D2 compared with those at DS2 but were down-regulated at D4 compared with those at DS4. Moreover, four carbonic anhydrase (CA) genes (*cynT*; Cannabis_sativa_newGene_18532, Cannabis_sativa_newGene_471, Cannabis_sativa_newGene_8219, and FN11569.1.g) were identified (Supplementary Table S7).

### DEGs involved in plant hormone signal transduction under drought stress

Plant hormone signal transduction plays a critical role in plant growth and abiotic stress tolerance. Among 65 DEGs associated with plant hormone signal transduction, 28 were regulated commonly to the two comparisons. These involved IAA, cytokinin (CTK), GA, ABA, brassinosteroid (BR), jasmonic acid (JA), and salicylic acid (SA) metabolic processes and signal transduction pathways. Auxin-related genes included one auxin transporter protein gene (*AUX1*; Cannabis_sativa_newGene_19036), two auxin-responsive protein genes (*IAA*; FN08651.1.g and FN27264.1.g), four SAUR family protein genes (*SAUR*; FN03063.1.g, FN03185.1.g, Cannabis_sativa_newGene_6221, and FN33877.1.g), and five auxin responsive GH3 gene family genes (*GH3*; FN00395.1.g, FN01004.1.g, FN15644.1.g, FN01628.1.g, and FN31094.1.g), all of which are involved in regulating the adaptive growth and development processes of plants, were induced by uniconazole in plants grown under drought stress. Four histidine kinase genes (*AHK2_3_4*; FN12857.1.g, FN18674.1.g, FN27301.1.g, and FN10311.1.g) and one two-component response regulator gene (*ARR-A*; FN19201.1.g), which are associated with biosynthesis and signal transduction of CKs, were induced by uniconazole in plants grown under drought stress. One DELLA protein (*DELLA*; Cannabis_sativa_newGene_9451), which is related to the signal transduction pathway of GA, was up-regulated by uniconazole in plants grown under drought stress. ABA-related genes including one protein phosphatase gene (*PP2C*; FN20151.1.g) and two serine/threonine-protein kinase genes (*SnRK2*; Cannabis_sativa_newGene_8004 and Cannabis_sativa_newGene_919) were down-regulated by uniconazole in plants grown under drought stress. Genes of *BSK* (FN03133.1.g), *CYCD3* (FN12036.1.g), *JAZ* (Cannabis_sativa_newGene_14430 and Cannabis_sativa_newGene_18071), *MYC2* (FN04245.1.g) were up-regulated, and two transcription factor *TGA* genes were down-regulated by uniconazole in plants grown under drought stress (Fig. 7c, Supplementary Table S8).

## Discussion

When plants are subjected to drought stress, total Chl (e.g., Chla, Chl b, and total Chl) and Car levels may decrease^24^. Drought stress inhibits the synthesis of Chl a/b^24^, affects light energy absorption, and transmission and conversion of photosystem I (PSI) and photosystem II (PSII) as well as decreases the photosynthetic rate^25^. In our study, Chl content decreased under drought stress in industrial hemp (Fig. 1a–d). Our finding that *P*_*N*_ was reduced in the leaves of industrial hemp seedlings under drought stress (Fig. 1e) was similar to that documented by Jhou et al.^26^. Reportedly, uniconazole application markedly improves contents of both Chl and Car and protects plants against drought^27^. Similar results were observed in this study (Fig. 1a–d). Many genes involved in porphyrin biosynthesis, such as those for glutamyl-tRNA reductase, protoporphyrinogen oxidase, and the Mg chelatase subunit, are regulated to provide better drought tolerance in plants^28^. Glutamyl-tRNA reductase, a key enzyme in plant porphyrin biosynthesis, affects Mg chelatase and enzymes involved in Chl synthesis^29^. Mg chelatase catalyses the first step of Chl synthesis^30^. Mg protoporphyrin IX monomethyl ester (oxidative) cyclase is a major enzyme involved in Chl biosynthesis, and induction of the corresponding genes improves Chl content and restores chloroplast structure^31^. Chlorophyll(ide) b reductase is a necessary enzyme for Chl b degradation, and inhibition of corresponding genes reduces Chl and Car degradation and promotes thylakoid grana retention^32^. In the present study, the expression of genes corresponding to glutamyl-tRNA reductase, Mg chelatase subunit, Mg protoporphyrin IX monomethyl ester (oxidative) cyclase, protochlorophyllide reductase, and Chlorophyll(ide) b reductase were regulated by uniconazole under drought stress (Fig. 6a, Supplementary Table S2). The differential expression of porphyrin and Chl metabolism pathway genes suggested that contents of Chl and Car were affected by uniconazole under drought stress (Fig. 1a–d). Some reports showed that under drought stress, plants treated with uniconazole had a higher *P*_N_ compared to that in untreated plants^15^ same to our study (Fig. 1e). Uniconazole application under drought stress can increase the levels of compounds involved in osmoregulation, lessen lipid peroxidation^16^, and protect cell integrity. These positive effects can improve absorption of light energy and thereby enhance photosynthesis capacity. Light harvesting Chl a/b-binding proteins (*LHCs*), as the most abundant membrane proteins, are involved in ABA signal transduction by regulating ROS (reactive oxygen species) homeostasis to improve tolerance to drought stress and grain yield^33^. In the present study, photosynthesis-antenna proteins pathway genes encoding *LHCs* were up-regulated by uniconazole under drought stress (Fig. 6b, Supplementary Table S3). These results suggest that uniconazole may modulate the drought resistance mechanisms of plants by changes in *LHC* gene expression. Photosystem II is a unique photosynthesis pigment-protein complex and a site for photosynthetic water oxidation^34^. The three photosystem II oxygen-evolving enhancer proteins and two photosystem II reaction centre subunits encoded by *psbQ*, *psbP*, *psb28*, and *psbW* are required for oxidation of water to O_2_ and thus are essential enzymes (Fig. 6c, Supplementary Table S4). Cytochrome c6 and plastocyanin are soluble electron carriers that transfer electrons to PSI^35^. In our study, one cytochrome c6 gene and one PC gene were up-regulated by uniconazole under drought stress. Moreover, uniconazole treatment under drought stress regulated seven PSI reaction centre subunit genes coding for proteins that catalysed the transfer of electrons from PC to FD through a series of electron transporters (Fig. 6c, Supplementary Table S4). FD receives electrons from PSI and transfers electrons to FD-NADP reductase (FNR), FNR induces the transfer of electrons from reduced FD to NADP^+^, and generates reduced NADPH for CO_2_ fixation and other chloroplast metabolic processes in the Calvin cycle^36^. Our study showed that four FD genes and two FD-NADP reductase genes were induced by uniconazole in plants grown under drought stress (Fig. 6c, Supplementary Table S4). The differential expression of photosynthetic pathway genes indicated that photosynthesis plays a significant role in the response to drought stress by uniconazole.

Carbon and nitrogen metabolisms, as the two most basic metabolic processes in plant physiology, affect the formation, transformation, and transportation of photosynthetic products^37^. Plants grown under drought stress are characterized by reduced Chl contents and decreased photosynthesis, which inhibits starch synthesis. In addition, starch was converted into soluble sugar to maintain cell osmotic pressure^38^. Some studies showed that the content of soluble sugar and sucrose increases but of starch content decreases^39^, which may be related to the increase of carbohydrate metabolism activity under drought stress. Analogous consequences were observed in our study: soluble sugar and sucrose content improved, whereas starch content decreased with prolonged drought stress (Fig. 2a–c). SPS, SS, S-AI, and NI are key enzymes involved in starch biosynthesis. Zahoor et al.^39^ reported an increase in SPS and SS activities but a decrease in S-AI activity in response to drought stress. In other reports, SS activity was lowered^40^ and SPS activity was enhanced^41^ by stress. These results were replicated in our studies, indicating that S-AI, NI, and SS activities were significantly reduced but SPS activity was increased in drought-treated plants (Fig. 2d–g). SS can catalyse both the synthesis and decomposition of sucrose. Considering our results, SS might play a role in sucrose decomposition. Improved SPS activity changed the distribution of carbon assimilates, resulting in increased contents of soluble sugar and sucrose, which are important for regulating cell osmotic pressure to resist drought stress^42^. Uniconazole markedly raised the soluble sugar content during waterlogging^43^, a similar finding was observed here (Fig. 2a). It was reported that exogenous spraying with uniconazole increased starch accumulation, starch granule content, and key enzyme activities involved in starch synthesis ^22^, same effects were noted in our study (Fig. 2b). An increase in sucrose content and a decrease in starch content were induced by drought stress, indicating that drought promoted the distribution of photosynthetic products to sucrose. Exogenous uniconazole application improved starch content, whereas it reduced the sucrose content of industrial hemp leaves under drought stress (Fig. 2b, c). A reasonable explanation is that uniconazole could enhance the photosynthetic capacity of drought-stressed industrial hemp leaves, which possibly adjusted the synthesis of sucrose and starch influenced by drought stress. Our results revealed that in plants under drought stress, uniconazole supplementation increased S-AI, NI, and SS activities compared with no supplementation (Fig. 2d–f), suggesting that uniconazole promotes sucrose decomposition and regulates photo-assimilation and translocation. SPS affects the distribution of photosynthetic products between starch and sucrose, and its activity is negatively correlated with starch accumulation and positively with sucrose accumulation^44^. We observed similar results: uniconazole decreased SPS activity, increased starch content, and reduced sucrose content under drought stress (Fig. 2b–g). Drought stress has been reported to influence SPS transcripts levels, indicating that SPS enzymes play central roles in starch and sucrose metabolism pathways^45^. In the present study, five SPS genes were regulated by uniconazole under drought stress (Supplementary Table S5). Previous studies have shown that sucrose synthase genes are regulated by drought stress, improving hyperosmotic stress tolerance of plants in relation to the accumulation of starch and sucrose metabolism proteins^46^. The present study described that two *SuSy* genes were differentially expressed in drought stress induced by uniconazole (Fig. 7a, Supplementary Table S6). In addition, *INV* (1), *scrK* (4), *bglX* (8), *GN4* (3), *glgC* (2), *PYG* (3), *GBE1* (2), *TPS* (7), *TREH* (3), and beta-amylase (1) genes were differentially expressed (Fig. 7a, Supplementary Table S6). The differential expression of these genes suggested that the starch and sucrose metabolic pathways were involved in the responses of industrial hemp leaves to drought stress under uniconazole treatment.

Nitrogen metabolism has great significances in plant growth, ecosystem structure and function^47^. Plants absorb nitrate nitrogen (NO_3_^−^) and ammonium nitrogen (NH_4_^+^) from soil, which are then converted into amino acids, proteins, and nucleotides by the key enzymes of NR, nitrite reductase, GS, GOGAT, and GDH^47^ that are crucial to nitrogen assimilation. Reportedly, the activities of NR, GS, and GOGAT in the functional leaves of plants were significantly reduced by drought stress, which affected the assimilation of NO_3_^−^ and NH_4_^+^, then inhibited nitrogen metabolism in the leaves of plants^40^. Our results were similar to these: NR, GS, and GOGAT activities of industrial hemp leaves were lower in drought stressed seedlings than in controls (Fig. 3a–c). Hessini et al.^48^ reported that GS levels decreased but GDH activity increased and proline levels improved, suggesting that GDH might be implicated in the synthesis of compatible solute under drought stress. In our results, the activity of GDH increased in drought stressed plants (Fig. 3d). Previous studies with uniconazole revealed that it has important roles in regulating levels of metabolites and promoting an increase in NR activity^49^. These results are similar to those seen in our study, where uniconazole showed increased NR activity under drought stress (Fig. 3a). Overexpression of the NR gene delayed the decrease in NR activity under drought stress, and the plants rapidly recovered N assimilation after rehydration^50^. In our study, one nitrate/nitrite transporter gene was up-regulated significantly by uniconazole under drought stress (Fig. 7b, Supplementary Table S7). Previous studies indicated that the regulation of a nitrate transporter gene implicated in N utilization decreased NO_3_^−^ accumulation of guard cells and enhanced drought tolerance of plants^51^. In plants under drought stress, exogenous uniconazole application enhanced GS activity compared with no application (Fig. 3b), which is consistent with the results of a previous study^52^. The GS/GOGAT pathway is the major system for utilizing NH_4_^+^ in plants, in our study, exogenous uniconazole application increased the activities of GS and GOGAT to relieve the toxic effect of NH_4_^+^ over-accumulation on plant seedlings by converting NH_4_^+^ to glutamate^53^. Previous studies showed that the transcription of glutamine synthetase genes and glutamate synthase genes in different tissues was up-regulated or down-regulated with changes in nitrogen levels^54^. Overexpression of glutamine synthetase *OsGS1;1* and *OsGS2* genes could strengthen the tolerance of plants to adverse abiotic stresses at the seedling stage in rice^55^. Lu et al.^56^ revealed that the suppression of glutamate synthase genes affected nitrogen assimilation in plants and had significant roles in carbon and nitrogen metabolism. In our studies, one glutamine synthetase gene (*glnA*) and two glutamate synthase genes (*GLT1*) were regulated by uniconazole under drought stress (Fig. 7b, Supplementary Table S7). These results revealed that uniconazole promoted the GS/GOGAT cycle, which is important in nitrogen metabolism. Reportedly, GS activity was reduced and GDH activity was increased under conditions of adversity stress, suggesting that glutamate and proline were produced by GDH^48,57^. Our study showed that DS plants exhibited lower GDH activity than D plants (Fig. 3d). Lightfoot et al.^58^ found that *gdhA* transgenic plants had improved seed germination rate, growth level, and grain biomass production and showed enhanced plant resistance by promoting nitrogen uptake^59^. In our results, four glutamate dehydrogenase genes were induced by uniconazole under drought stress (Fig. 7b, Supplementary Table S7). In addition, four CA genes (*cynT*) were differentially expressed under drought stress treated with uniconazole (Fig. 7b, Supplementary Table S7), which may catalyse the conversion between CO_2_ and HCO_3_^−^, and play important functions in photosynthesizing cells of plants under environmental stress. The differential expression of nitrogen metabolism pathway genes revealed that nitrogen metabolism had an effect on plants under drought stress treated with uniconazole.

A series of changes in plant endogenous hormones under drought stress reflects the adaptability of plants to stress. Moreover, drought resistance in plants is achieved by the complex coordination of various hormones, rather than by a single hormone. ABA and ZT, as critical endogenous hormones, take part in regulating the abiotic stress response. Some studies showed that drought stress substantially enhanced endogenous hormone ABA level but reduced ZT content, indicating that ABA and ZT jointly controlled plant photosynthetic performance in response to drought stress^60^. Our results similarly showed that ABA content was increased but ZT level was decreased under drought stress (Fig. 4a, d). Some reports revealed that ABA and ZT levels significantly increased in plants grown under abiotic stress and treated with uniconazole^14^, promoting the accumulation of proline and abiotic stress-related amino acids^13^. We noticed that uniconazole application notably increased ABA and ZT contents under drought stress for 4 days (Fig. 4a, d). Previous studies showed that histidine kinases (AHKs) positively affected drought stress responses through the ABA signalling pathway^61^. Two-component response regulator genes are also employed by plants for stress adaptation^62^. A previous study revealed that ABA signalling key kinases interacted with the CTK signalling regulator ARR in response to drought stress^63^. Liu et al.^21,22^ reported that uniconazole regulated AHKs and type-B ARRs to affect Chl degradation. In our studies, histidine kinase genes (*AHK2_3_4*) and two-component response regulator gene (*ARR-A*) were induced by uniconazole under drought stress (Fig. 7c, Supplementary Table S8). Protein phosphatase genes have been isolated and cloned from plants and may play key roles in signal transduction in drought stress^64^. Serine/threonine-protein kinases are the key regulatory factors of ABA signalling pathways, which are involved in abiotic stress adaptation in plants^65^. In our studies, protein phosphatase (*PP2C*) and serine/threonine-protein kinase (*SnRK2*) were down-regulated by uniconazole as an adaptation to drought stress (Fig. 7c, Supplementary Table S8). Drought stress blocks IAA signal transduction, decreases IAA biosynthesis, and markedly reduces IAA content^66^. In our study, IAA content was decreased with prolonged drought stress (Fig. 4b). Exogenous uniconazole treatment increased IAA content compared with no treatment in seedlings under drought stress (Fig. 4b). A logical explanation is that uniconazole inhibited IAA oxidase activity under drought stress, thus increasing IAA concentration. Similar conditions have been identified wherein uniconazole-treated plants had increased IAA content under water deficit stress^15^. It has been reported that auxin transporter genes (*AUX*/*LAX*)^67^, auxin-responsive protein (*IAA* and *SAUR*)^68^, and indole-3-acetic acid-amido synthetase gene (*GH3*)^69^ are involved in the auxin signalling pathway and play important roles in drought tolerance. In the present study, auxin-related genes (*AUX1*, *IAA*, *SAUR*, and *GH3*) were induced by uniconazole in response to drought stress (Fig. 7c, Supplementary Table S8). Gibberellin plays a key role in different stages of plant growth, such as seed germination, internode elongation, and flower and fruit formation and is involved in regulating abiotic stress processes^70^. The GA_3_ level decreased under drought stress, restricting the growth of plants^15^. In our studies, the content of endogenous GA_3_ reduced during the final d of drought stress (Fig. 4c). Uniconazole is an inhibitor of gibberellin biosynthesis, reports also found that endogenous GA_3_ content decreased in uniconazole-treated plants^21,22^ under stress^15^. These results were similar to those obtained in our study, where supplemental uniconazole under drought stress decreased the GA_3_ content (Fig. 4c). DELLA proteins, members of the plant-specific GRAS family, negatively regulate plant growth^71^. In previous studies, abiotic stress promoted the accumulation of DELLA proteins and then reduced endogenous hormone GA levels to inhibit plant growth and enhance plant tolerance to stress^70^. However, a study had shown that the overexpression of DELLA proteins (S-*della*) increased stomatal sensitivity to ABA and reduced water loss^72^. Uniconazole affected not only the GA biosynthesis pathway but also related genes in this pathway^23^. A previous study showed that uniconazole regulated the expression of DELLA proteins to affect plant growth^22^. In our study, one DELLA protein was up-regulated by uniconazole under drought stress (Fig. 7c, Supplementary Table S8). In addition, *BSK* (1) and *CYCD3* (1) genes involved in brassinosteroid biosynthesis; *JAZ* (2) and *MYC2* (1) genes involved in jasmonic acid metabolism; and two transcription factor TGA genes involved in salicylic acid biosynthesis were differentially expressed (induced) by uniconazole (Fig. 7c, Supplementary Table S8). Numerous genes were differentially expressed, showing that plant hormone signal transduction pathways are involved in the response of industrial hemp leaves in plants grown under drought stress and treated with uniconazole.

**Figure 8:**
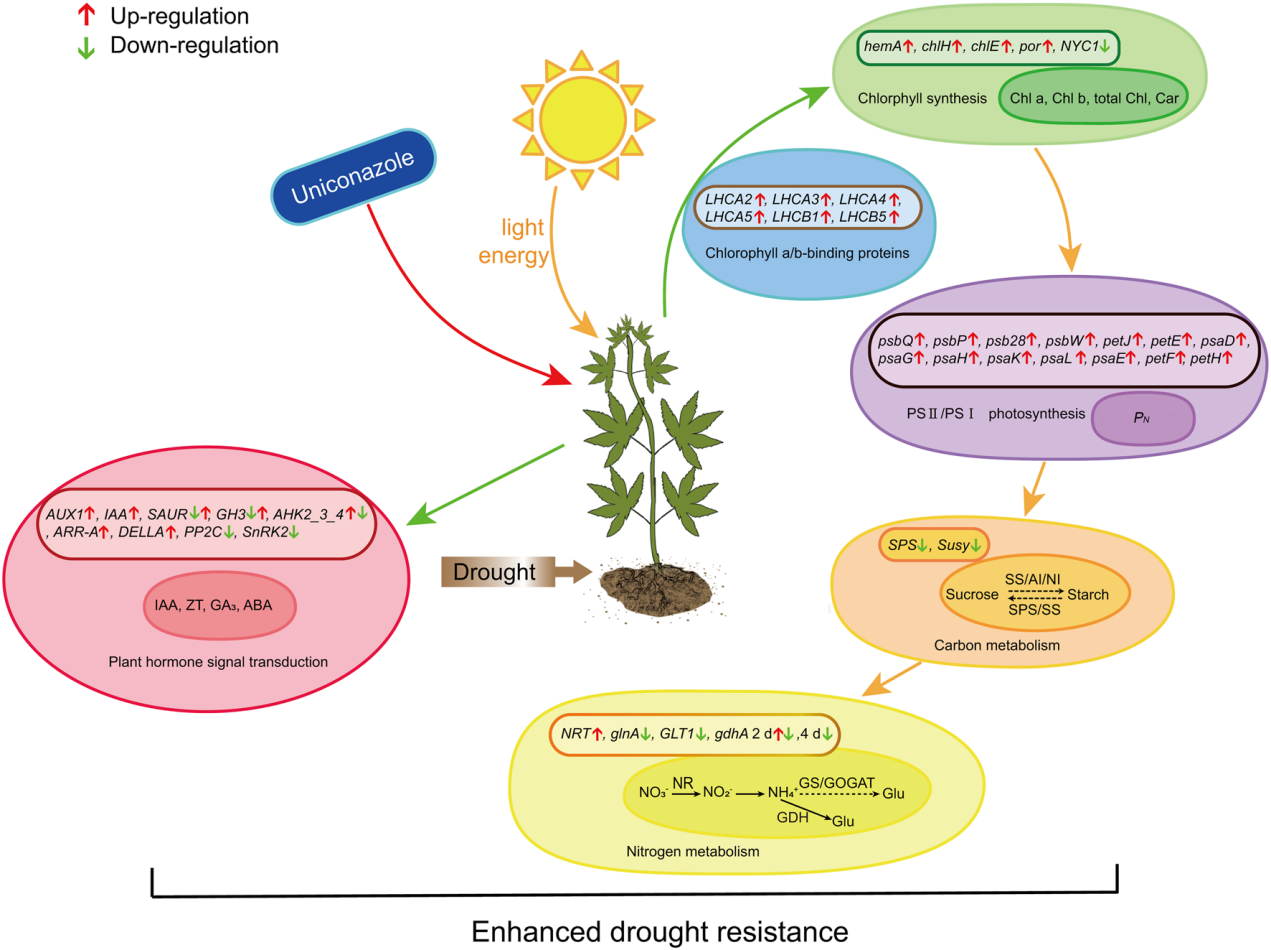
A hypothetical model of uniconazole on physiological and genome-wide gene expression profiling in industrial hemp seedlings under drought stress. The large ellipse indicates the whole metabolic pathway, the small ellipse represents physiological index, box means differentially expressed genes. The image was drawn by using Adobe Illustrator CC2020 (Adobe Systems Inc, San Jos, CA, USA).

Based on the above discussion, a hypothetical model of the effects of uniconazole on physiology and genome-wide gene expression in industrial hemp seedlings grown under drought stress is presented in Fig. 8.

## Methods

### Plant material and seedling treatments

‘Hanma No. 2’, an industrial hemp cultivar, was used in this study. The variety was bred and served at Daqing Branch of Heilongjiang Academy of Sciences (Daqing, China), and the seeds are available from the first author on reasonable request. Germinated seeds were sown in plastic pots with a height of 16 cm, mouth diameter of 20 cm, and bottom diameter of 12.5 cm filled with 2:1 (v/v) grass peat and sand. The properties of grass peat were as follows: maximum field capacity (FC), 46%; pH 6.3; organic matter content, 20.4%; available P, 13.7 mg/kg; NH_4_^+^, 315.3 mg/kg; and available K, 206 mg/kg. In total, 15 seedlings per pot were maintained after seed germination. Industrial hemp seedlings were grown using the weighing method, and an appropriate amount of water was added to maintain 70% of FC until the trifoliate stage in the mobile canopy. At the three-leaf stage, half of potted hemp plants (40 pots) were sprayed with 40 mg L^−1^ uniconazole solution^73^, and the others were sprayed with distilled water. At 48 h after spraying, the plants were subjected to drought stress for 0, 2, 4, 6, and 8 days. D and DS were used to represent drought stress after spraying with distilled water and drought stress after spraying with uniconazole, respectively. The soil moisture content at 0, 2, 4, 6, and 8 days was maintained at 32%, 28%, 23%, 18%, and 14%, respectively. For physiological experiments, the fully expanded third pair of leaves from the plant base was collected at each treatment; one biological repeat had twenty leaves, and four biological repeats were used. For endogenous hormone levels and RNA-seq, the completely expanded third pair of leaves from the base of plants subjected to drought stress for 2 and 4 days was harvested, and three biological repeats were used for D and DS treatments. The samples were frozen in liquid nitrogen immediately and stored at – 80 °C.

### Chl content and net photosynthetic rate

Chl, including Chl a, Chl b, and total Chl, and Car contents were measured as reported by Arnon^74^ with a few modifications. Fresh leaves without veins (0.1 g) were soaked in ethanol and acetone (v/v, 1:1) until the leaves became colourless. Absorbances of the extracts were recorded at 470, 645, and 663 nm.

*P*_*N*_ of the third pair of leaves was measured between 9:00 and 11:00 am using a portable photosynthesis system (LI-6400 XT, LI-COR, Inc. Lincoln, NE, USA) under light intensity of 1000 µmol m^−2^ s^−1^.

### Carbon metabolism-related enzyme activities

Soluble sugar content was measured using the procedure reported by Spiro^75^ with a slight modification. Leaves (0.3 g) were cut and placed in a tube, to which 10 mL of distilled water was added. The tube was boiled for 30 min and the extract solution was filtered into a 25 mL volumetric flask. Ethyl anthrone acetate (0.5 mL), H_2_SO_4_ (5 mL), and distilled water (1.5 mL) were mixed with the leaf extract (0.5 mL). The mixture was then boiled for 1 min, and the absorbance was recorded at 620 nm after cooling.

Starch content was determined by the method reported by Yemm and Willis^76^. Leaves (0.1 g) were ground and extracted with 80% ethanol, then centrifuged to remove supernatant. Distilled water was added to the sediment, and the slurry was gelatinized in a boiling water bath for 15 min. Perchloric acid (9.2 M) was used to extract the starch. Distilled water (1.5 mL) and anthrone reagent (anthrone dissolved in concentrated H_2_SO_4_, 6.5 mL) were quickly mixed with the extracted solution (1 mL), and colour development was observed for 15 min. After cooling, the absorbance was measured at 620 nm.

To determine sucrose content, 100 μL of supernatant was mixed with 50 μL NaOH (2 M) and boiled for 5 min. Next, 700 μL 30% HCl and 200 μL 0.1% resorcinol were added, and the mixture was boiled for 30 min. After cooling, the absorbance was monitored at 480 nm using distilled water as blank. Sucrose was extracted using assay kits from Cominbio Company Ltd. (Suzhou, China).

For determination of S-AI, NI, SS, and SPS activities, leaves were homogenized with 0.1 M phosphoric acid buffer (pH 7.5), 5 mM MgCl_2_, 1 mM EDTA, 0.1% mercaptoethanol, and 0.1% TritonX-100 mixture in an ice bath, followed by centrifugation at 12,000×*g* for 15 min to collect the supernatant. The enzyme activities of S-AI, NI, SS, and SPS were measured using enzyme kits (Cominbio, Suzhou, China) according to manufacturer’s instructions. The activities of S-AI, NI, and SS were expressed as microgram (product reducing sugar) per minute per gram^77^. One SPS activity unit was defined as the production of 1 μg sucrose per min per gram of fresh leaves^77^.

### Nitrogen metabolism-related enzyme activities

The activities of nitrogen metabolism-related enzymes NR, GS, GOGAT, and GDH in fresh leaves were determined using test kits (Cominbio, Suzhou, China). For NR activity, the absorbance was recorded at 340 nm using distilled water as a blank. One unit of NR activity was defined as the reduction of 1 μmol NADH per hour per gram of fresh leaves^78^. GS activity was analyzed using a GS detection kit, and an enzyme activity unit was calculated as the production of 1 μmol γ-glutamylhydroxamic acid per hour per gram of fresh leaves per millilitre of the reaction system at 540 nm^79^. GOGAT and GDH activities were monitored using the absorbance at 340 nm, and the consumption of 1 nmol of NADH per minute per gram tissue was defined as one unit enzyme activity^79^.

### Endogenous levels of ABA, IAA, GA_3_, and ZT

Leaves (0.1 g) were ground in liquid nitrogen; then added into a 1 mL mixture of methanol, water, and acetic acid; and extracted overnight at 4 °C. The extracts were centrifuged at 8000×*g* for 10 min, and the supernatant was dried to the water phase with nitrogen. After adjusting the pH to 2–3 with citric acid, the solution was extracted three times with ethyl acetate. The ethyl acetate phases were combined and dried using a stream of nitrogen and then made up to a constant volume with methanol. The solution was filtered through a 0.22-μm organic filter and assessed using a liquid chromatography-tandem mass spectrometry system. The levels of the endogenous hormones ABA, IAA, GA_3_, and ZT in the leaves of hemp plants grown under D and DS for 2 and 4 days were analyzed using the method reported by Farrow and Emery^80^.

### RNA-seq library preparation and sequencing

Total RNA was extracted from the fully developed third leaves of D2, D4, DS2, and DS4 using TRIzol reagent (Invitrogen, Carlsbad, CA, USA), and residual DNA was eliminated with DNase I (Fermentas, Vilnius, Lithuania). RNA concentration and quality were evaluated using a Nanophotometer Spectrophotometer (Implen, CA, United States) and an RNA Nano 6000 assay kit for the Agilent Bioanalyzer 2100 system (Agilent Technologies, Santa Clara, CA, United States). The RNA sequencing library was constructed following the manufacture’s recommendation by using NEBNext Ultra RNA Library Prep Kit for Illumina (NEB, Ipswich, MA, USA). Equal amounts of total RNA from each sample were pooled together. Then the mRNAs were purified from total RNA by poly-T oligo-attached VAHTS mRNA Capture Beads (Vazyme BioTech, Nanjing, China). The mRNAs were fragmented to avoid priming bias when synthesizing cDNA by using divalent cations under elevated temperature in NEBNext First Strand Synthesis Reaction Buffer. First-strand cDNAs were synthesized using random hexamer primer and M-MuLV Reverse Transcriptase (RNase H-). Second-strand cDNAs were subsequently synthesized with DNA Polymerase I and RNase H. Double-stranded cDNAs were purified by VATHS magnetic DNA clean beads (Vazyme BioTech). Then the End Repair Reaction Buffer and End Prep Enzyme Mix were added into the purified products for end repair and adenine (A) addition of 3'-end. The adaptor-ligated cDNAs were then added with USER Enzyme (NEB) for adaptor cleavage. Subsequently, library size selection was conducted using magnetic purification beads (Vazyme BioTech) to capture DNA fragments in a given size range, remove unwanted DNA fragments, and remove contaminants such as enzymes. Then PCR was performed with Phusion High-Fidelity DNA Polymerase, Universal PCR Primers and Index (X) Primer. At last, PCR products were purified with Vazyme VATHS DNA clean beads and library quality was assessed on the Agilent Bioanalyzer 2100 system. The RNA-seq library preparations were sequenced on an Illumina HiseqX-ten platform (San Diego, CA, USA) at Biomarker Technologies Co., Ltd. (Beijing, China), following the manufacturer’s instructions (Illumina).

### Date processing and identification of DEGs

A large amount of raw data (raw reads) was screened with in-house perl scripts to obtain clean data (clean reads) by removing reads containing adapter and poly-N, and low quality reads. The GC content, Q30 and sequence duplication levels were calculated. High-quality clean reads were mapped to the *C. sativa* L. reference genome sequence with HISAT2^81^.

Gene expression levels were represented using the fragments per kilobase of exon per million mapped read (FPKM) value of each transcript. The abundance of the same transcripts of D2_vs_DS2 and D4_vs_DS4 samples were compared to identify DEGs using DESeq2^82^. This study compared the effects of uniconazole treatment on DEGs in the leaves of industrial hemp grown for 2 and 4 days under drought stress using fold change ≥ 2 and false discovery rate < 0.01 as the screening criteria.

### KEGG pathway enrichment analysis

KEGG pathway enrichment analysis was performed using the KEGG pathway mapping tool (http://www.genome.jp/kegg)^83–85^ to analyze significantly enriched metabolic pathways or signal transduction pathways of DEGs.

### qRT-PCR of mRNAs

Twenty-one genes showing different expression levels in RNA-Seq analyses were selected to confirm the results of RNA-seq with the method of quantitative real-time PCR (qRT-PCR) analyses. Total RNA was extracted and purified according to the above method. Subsequently, 1 μg of purified RNA was reverse-transcribed using the FastKing RT Kit (Tiangen BioTek, Beijing, China) to produce cDNA. The GAPDH gene was selected as the reference gene. The qRT-PCR system contained Power qPCR PreMix (Genecopoeia, Rockville, MD, USA), cDNA, and the forward and reverse gene-specific primers. The reaction procedure included 40 cycles of a denaturation step at 95 °C for 10 s, following by an annealing step at 60 °C for 40 s. The experiment consisted of three repetitions. Gene-specific primers were designed by Primer Premier 5 software (Premier Biosoft International, Palo Alto, CA, USA) and listed in Supplementary Table S9.

### Data analysis

All experimental data were analyzed by analysis of variance using SPSS software version 20.0 (IBM Inc., Chicago, IL, USA) and presented as mean ± standard errors. Duncan’s multiple range test (*P* < 0.05) was applied to detect differences between means. All graphs were generated using OriginPro 9.1 software (OriginLab, Northampton, MA, USA).

The original RNA sequencing data were submitted to NCBI’s Gene Expression Omnibus (http://www.ncbi.nih.gov/geo/) under series number PRJNA635553.

All methods were performed in accordance with the relevant national or international guidelines/regulations/legislation.

## Supplementary Information

Supplementary Figure S1.

Supplementary Tables.
