# Supplementary material for: Physiological and transcriptome analyses for assessing the effects of exogenous uniconazole on drought tolerance in hemp (Cannabis sativa L.)
Source: Sci Rep. 2021 Jul 14;11:14476. doi: 10.1038/s41598-021-93820-6 (PMC8280108; doi:10.1038/s41598-021-93820-6)
Supplement: Supplementary file 1 — Supplementary Figure S1. [file 41598_2021_93820_MOESM1_ESM.pdf]

TITLE:

Physiological and Transcriptome Analyses for Assessing the Effects of Exogenous Uniconazole on Drought Tolerance in Hemp (*Cannabis sativa* L.)

AUTHORS:

Ying Jiang [1,2], Yufeng Sun [2], Dianfeng Zheng [1,3,4], Chengwei Han [2], Kun Cao [1,2], Lei Xu [1], Shuxia Liu [1,2], Yanyong Cao [5,] & Naijie Feng [1,3,4,]

AFFINITIONS

[1]College of Agronomy, Heilongjiang Bayi Agricultural University, Daqing 163316, China. [2]Daqing Branch of Heilongjiang Academy of Sciences, Daqing 163319, China. [3]College of Coastal Agricultural Sciences, Guangdong Ocean University, Zhanjiang, 524088, China. [4]Shenzhen Research Institute of Guangdong Ocean University, Shenzhen, Guangdong, 518108, China. [5]Institute of Cereal Crops, Henan Academy of Agricultural Sciences, Zhengzhou 450002, China. Correspondence and requests for materials should be addressed to N. F. (email: byndfnj@126.com) or Y. C. (email: yanyongcao@126.com).

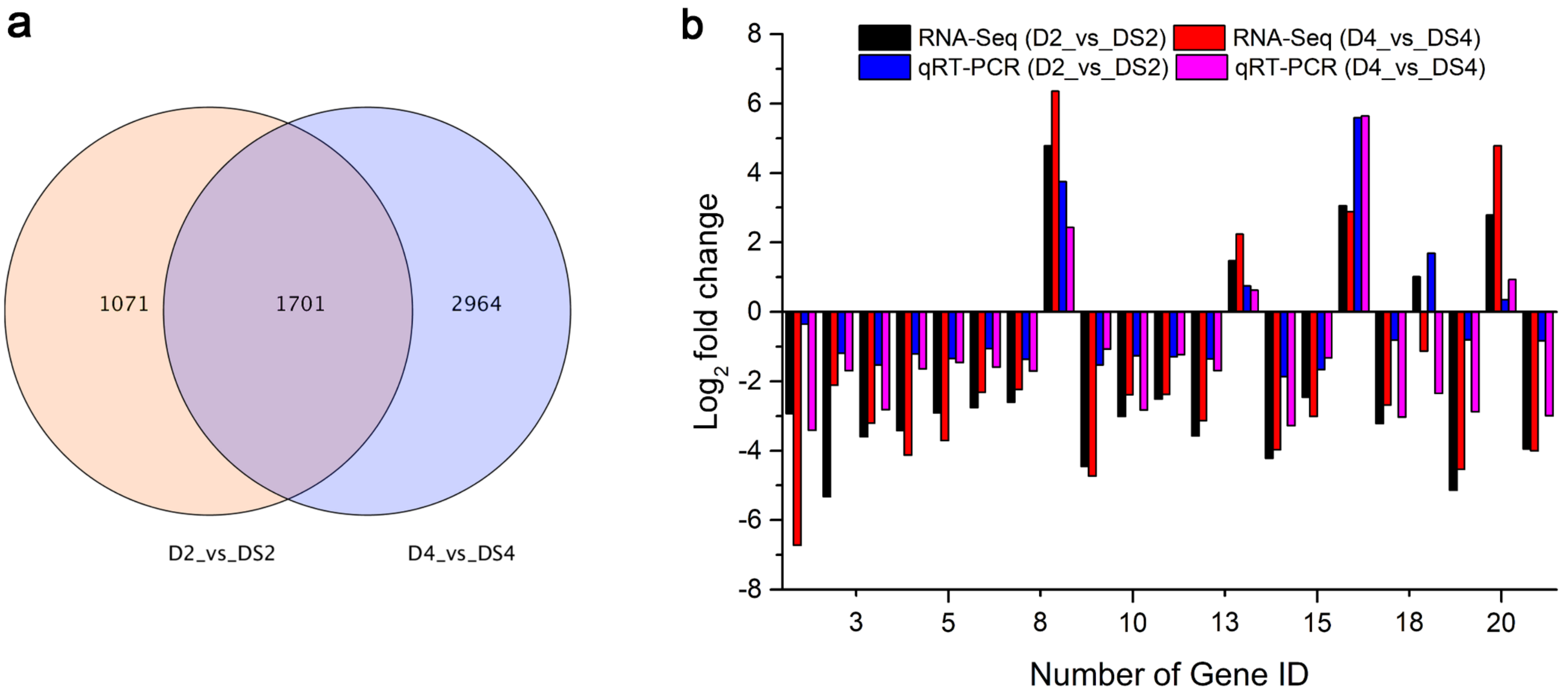

Supplementary Figure S1. Effect of uniconazole on genome-wide gene expression profiling of industrial hemp seedlings under drought stress. Uniconazole-induced venn diagram of the differentially expressed genes for 2 and 4 days under drought stress. The intersection was the number of common differentially expressed genes after different treatment days (a). qRT-PCR validation of 21 DEGs. Expression changes of 21 genes of D and DS was analyzed by qRT-PCR and compared with RNA-Seq data (b). D2_vs_DS2 means drought stress for 2 days / drought stress treated with uniconazole for 2 days; D4_vs_DS4 means drought stress for 4 days / drought stress treated with uniconazole for 4 days
