# Supplementary material for: Physiological and transcriptome analyses for assessing the effects of exogenous uniconazole on drought tolerance in hemp (Cannabis sativa L.)
Source: Sci Rep. 2021 Jul 14;11:14476. doi: 10.1038/s41598-021-93820-6 (PMC8280108; doi:10.1038/s41598-021-93820-6)
Supplement: Supplementary file 2 — Supplementary Tables. [file 41598_2021_93820_MOESM2_ESM.pdf]

TITLE:

Physiological and Transcriptome Analyses for Assessing the Effects of Exogenous Uniconazole on Drought Tolerance in Hemp (*Cannabis sativa* L.)

AUTHORS:

Ying Jiang [1,2], Yufeng Sun [2], Dianfeng Zheng [1,3,4], Chengwei Han [2], Kun Cao [1,2], Lei Xu [1], Shuxia Liu [1,2], Yanyong Cao [5,] & Naijie Feng [1,3,4,]

AFFINITIONS

[1]College of Agronomy, Heilongjiang Bayi Agricultural University, Daqing 163316, China. [2]Daqing Branch of Heilongjiang Academy of Sciences, Daqing 163319, China. [3]College of Coastal Agricultural Sciences, Guangdong Ocean University, Zhanjiang, 524088, China. [4]Shenzhen Research Institute of Guangdong Ocean University, Shenzhen, Guangdong, 518108, China. [5]Institute of Cereal Crops, Henan Academy of Agricultural Sciences, Zhengzhou 450002, China. Correspondence and requests for materials should be addressed to N. F. (email: byndfnj@126.com) or Y. C. (email: yanyongcao@126.com).

Supplementary Table S1. Evaluation statistics of sequencing data

Sample	Total Reads	Clean reads	Clean bases	Mapped Reads	GC Content	%≥Q30
D2-1	41,363,184	20,681,592	6,175,384,072	30,930,443 (74.78%)	45.26%	94.36%
D2-2	39,506,934	19,753,467	5,894,484,272	29,669,323 (75.10%)	45.25%	95.17%
D2-3	39,969,310	19,984,655	5,962,931,224	30,045,508 (75.17%)	45.26%	95.01%
D4-1	40,979,786	20,489,893	6,124,499,708	30,064,442 (73.36%)	44.66%	91.73%
D4-2	38,966,386	19,483,193	5,816,373,684	28,658,169 (73.55%)	44.65%	92.50%
D4-3	38,948,046	19,474,023	5,821,193,082	28,815,286 (73.98%)	44.83%	92.72%
DS2-1	40,788,036	20,394,018	6,090,390,944	30,552,542 (74.91%)	45.22%	92.78%
DS2-2	41,995,488	20,997,744	6,271,485,606	30,699,975 (73.10%)	45.58%	92.64%
DS2-3	41,727,142	20,863,571	6,226,507,656	30,397,266 (72.85%)	45.44%	92.59%
DS4-1	42,484,078	21,242,039	6,330,791,892	31,710,236 (74.64%)	45.23%	95.72%
DS4-2	54,906,362	27,453,181	8,184,300,418	42,250,286 (76.95%)	45.41%	95.83%
DS4-3	44,609,406	22,304,703	6,649,827,682	33,376,699 (74.82%)	45.48%	95.77%

Supplementary Table S2. Uniconazole-induced expression patterns of 11 porphyrin and chlorophyll metabolism genes under drought stress

	D2_vs_DS2	D4_vs_DS4	
Gene ID	Log_2 fold change	Log_2 fold change	Annotation
Cannabis_sativa_newGene_12230	-2.05	-1.26	Pheophorbide a oxygenase
Cannabis_sativa_newGene_2056	-1.44	-2.22	Chlorophyll(ide) b reductase
Cannabis_sativa_newGene_6391	1.49	1.37	Magnesium-protoporphyrin IX monomethyl ester (oxidative) cyclase
Cannabis_sativa_newGene_6392	1.56	1.67	Magnesium-protoporphyrin IX monomethyl ester (oxidative) cyclase
Cannabis_sativa_newGene_6970	-1.47	-1.93	Chlorophyll(ide) b reductase
FN07522.1.g	1.08	1.51	Protochlorophyllide reductase
FN09070.1.g	3.05	2.88	Chlorophyllase
FN10979.1.g	3.84	3.44	Chlorophyllase
FN16017.1.g	1.3	1.24	Magnesium chelatase subunit H
FN25584.1.g	-1.53	-2.3	Chlorophyll(ide) b reductase
FN36173.1.g	2.13	1.13	Glutamyl-tRNA reductase

Supplementary Table S3. Uniconazole-induced expression patterns of 7 photosynthesis-antenna proteins genes under drought stress

Gene ID	D2_vs_DS2	D4_vs_DS4	Annotation
	Log2 fold change	Log2 fold change	
Cannabis_sativa_newGene_22639	1.43	1.35	Light-harvesting complex I chlorophyll a/b binding protein 3
FN00679.1.g	4.36	2.49	Light-harvesting complex II chlorophyll a/b binding protein 1
FN08854.1.g	1.46	1.72	Light-harvesting complex I chlorophyll a/b binding protein 2
FN11590.1.g	1.47	1.98	Light-harvesting complex II chlorophyll a/b binding protein 1
FN20197.1.g	1.06	1.01	Light-harvesting complex I chlorophyll a/b binding protein 5
FN20752.1.g	1.57	1.6	Light-harvesting complex I chlorophyll a/b binding protein 4
FN25278.1.g	1.66	1.44	Light-harvesting complex II chlorophyll a/b binding protein 5

Supplementary Table S4. Uniconazole-induced expression patterns of 20 photosynthesis genes under drought stress

Gene ID	D2_vs_DS2	D4_vs_DS4	Annotation
	Log_2 fold change	Log_2 fold change	
Cannabis_sativa_newGene_11217	1.03	1.57	Cytochrome c6
Cannabis_sativa_newGene_11948	1.78	1.18	Photosystem I subunit IV
Cannabis_sativa_newGene_18499	1.07	1.07	Ferredoxin-NADP reductase
Cannabis_sativa_newGene_18790	1.13	1.01	Ferredoxin-NADP reductase
Cannabis_sativa_newGene_2824	1.17	1.52	Ferredoxin
Cannabis_sativa_newGene_3177	1.28	1.11	Oxygen-evolving enhancer protein 2
Cannabis_sativa_newGene_8056	1.89	1.3	Photosystem I reaction center subunit IV B
FN00583.1.g	3.1	2.26	Ferredoxin
FN03578.1.g	1.16	1.65	Ferredoxin
FN05836.1.g	1.56	1.49	Photosystem I subunit VI
FN09237.1.g	1.64	1.11	Photosystem I subunit V
FN12930.1.g	2.64	2.15	Photosystem I subunit X
FN15653.1.g	1.88	1.57	Ferredoxin
FN19968.1.g	1.48	1.4	Photosystem I subunit II
FN22627.1.g	1.4	1.54	Plastocyanin
FN25592.1.g	1.17	1.2	Photosystem I subunit XI
FN28964.1.g	2.16	1.63	Photosystem II 13kDa protein
FN29818.1.g	1.15	1.12	Photosystem II oxygen-evolving enhancer protein 2
FN35311.1.g	1.95	2.17	Photosystem II PsbW protein
FN36957.1.g	1.96	1	Photosystem II oxygen-evolving enhancer protein 3

Supplementary Table S5. Uniconazole-induced expression patterns of 5 sucrose-phosphate synthase genes in starch and sucrose metabolism pathway under drought stress

Gene ID	D2_vs_DS2<br>Log_2 fold change	D4_vs_DS4<br>Log_2 fold change	Annotation
FN06206.1.g	-0.68	-1.03	Sucrose-phosphate synthase
FN08251.1.g	-0.78	-1.13	Sucrose-phosphate synthase
FN15293.1.g	-1.04	-0.81	Sucrose-phosphate synthase
FN19565.1.g	-0.71	-1.05	Sucrose-phosphate synthase
FN33647.1.g	-0.68	-1.18	Sucrose-phosphate synthase

Supplementary Table S6. Uniconazole-induced expression patterns of 39 starch and sucrose metabolism genes under drought stress

Gene ID	D2_vs_DS2 Log$_2$FC	D4_vs_DS4 Log$_2$FC	Annotation
Cannabis_sativa_newGene_11111	1.03	1.23	Glycogen phosphorylase
Cannabis_sativa_newGene_11739	1.25	1.59	Fructokinase
Cannabis_sativa_newGene_1196	1.98	2.32	Alpha-trehalase
Cannabis_sativa_newGene_1359	-1.31	-2.46	Trehalose 6-phosphate synthase/phosphatase
Cannabis_sativa_newGene_14442	-1.6	-1.23	Beta-amylase
Cannabis_sativa_newGene_15537	-2.76	-2.32	Beta-glucosidase
Cannabis_sativa_newGene_15584	2.59	1.26	Beta-glucosidase
Cannabis_sativa_newGene_16510	-2.61	-2.24	Beta-glucosidase
Cannabis_sativa_newGene_17325	1.08	1.27	Glucose-1-phosphate adenylyltransferase
Cannabis_sativa_newGene_18682	-3.01	-2.39	Sucrose synthase
Cannabis_sativa_newGene_19025	1.44	-2.86	Beta-glucosidase
Cannabis_sativa_newGene_2041	1.4	1.44	Fructokinase
Cannabis_sativa_newGene_20881	-2.51	-2.38	Beta-glucosidase
Cannabis_sativa_newGene_21583	2.43	1.16	Beta-glucosidase
Cannabis_sativa_newGene_22098	-1.55	-2.34	Trehalose 6-phosphate synthase/phosphatase
Cannabis_sativa_newGene_3872	1.01	1.47	Glucose-1-phosphate adenylyltransferase
Cannabis_sativa_newGene_4716	-1.41	-1.27	Trehalose 6-phosphate synthase/phosphatase
Cannabis_sativa_newGene_6352	-1.26	-1.01	UDP-glucuronic acid decarboxylase
Cannabis_sativa_newGene_7682	2.99	1.5	Beta-glucosidase
Cannabis_sativa_newGene_7780	1.27	1.68	Fructokinase
Cannabis_sativa_newGene_8314	-1.05	-1.74	Acid beta-fructofuranosidase
FN00010.1.g	1.46	1.67	Glucan endo-1,3-beta-glucosidase
FN00198.1.g	1.16	1.29	Glucan endo-1,3-beta-glucosidase
FN08139.1.g	-2.46	-3.01	Beta-glucosidase
FN09580.1.g	1.76	2.39	Glucan endo-1,3-beta-glucosidase
FN12624.1.g	-2.79	-2.29	Sucrose synthase
FN15223.1.g	1.94	1.72	Alpha-trehalase
FN17900.1.g	1.89	3.28	1,4-alpha-glucan-branching enzyme
FN21504.1.g	1.85	2.08	Alpha-trehalase
FN22315.1.g	2.09	2.89	Glycogen phosphorylase
FN24873.1.g	-2.04	-3.43	Trehalose 6-phosphate synthase/phosphatase
FN25057.1.g	-1.2	-2.1	Fructokinase
FN25284.1.g	-1.38	-2.24	Trehalose 6-phosphate synthase/phosphatase
FN28846.1.g	1.08	1.9	Pectinesterase
FN30251.1.g	-1.27	-2.04	Trehalose 6-phosphate synthase/phosphatase
FN33579.1.g	2.79	4.78	1,4-alpha-glucan-branching enzyme
FN35530.1.g	-1.47	-2.11	Trehalose 6-phosphate synthase/phosphatase
FN36185.1.g	-2.45	-1.98	Pectinesterase
FN37634.1.g	2.08	2.62	Glycogen phosphorylase

Supplementary Table S7. Uniconazole-induced expression patterns of 12 nitrogen metabolism genes under drought stress

Gene ID	D2_vs_DS2	D4_vs_DS4	Annotation
	Log_2 fold change	Log_2 fold change	
Cannabis_sativa_newGene_10837	-1.54	-1.95	Glutamate synthase (NADH)
Cannabis_sativa_newGene_1215	-3.6	-3.21	Glutamate dehydrogenase ($NAD(P)^+$)
Cannabis_sativa_newGene_18532	2.18	1.33	Carbonic anhydrase
Cannabis_sativa_newGene_23402	-3.58	-3.14	Glutamate dehydrogenase ($NAD(P)^+$)
Cannabis_sativa_newGene_4468	1.8	2.42	Nitrate/nitrite transporter
Cannabis_sativa_newGene_471	2	1.3	Carbonic anhydrase
Cannabis_sativa_newGene_6112	1.09	-1.08	Glutamate dehydrogenase ($NAD(P)^+$)
Cannabis_sativa_newGene_8219	3.43	2.65	Carbonic anhydrase
FN06032.1.g	-1.35	-1.85	Glutamate synthase (NADH)
FN11569.1.g	-1.68	-3.01	Carbonic anhydrase
FN22131.1.g	-1.15	-1.95	Glutamine synthetase
FN28192.1.g	1.01	-1.13	Glutamate dehydrogenase ($NAD(P)^+$)

Supplementary Table S8. Uniconazole-induced expression patterns of 28 plant hormone signal transduction genes under drought stress

	D2_vs_DS2	D4_vs_DS4	
Gene ID	Log_2 fold change	Log_2 fold change	Annotation
Cannabis_sativa_newGene_14430	1.68	1.51	Jasmonate ZIM domain-containing protein
Cannabis_sativa_newGene_18071	1.64	1.47	Jasmonate ZIM domain-containing protein
Cannabis_sativa_newGene_19036	1.93	1.19	Auxin transporter protein
Cannabis_sativa_newGene_21556	-1.05	-1.77	Transcription factor TGA
Cannabis_sativa_newGene_6221	2.17	1.94	SAUR family protein
Cannabis_sativa_newGene_8004	-1.8	-1.02	Serine/threonine-protein kinase SRK2
Cannabis_sativa_newGene_919	-1.56	-1.14	Serine/threonine-protein kinase SRK2
Cannabis_sativa_newGene_9451	1.47	2.24	DELLA protein
FN00395.1.g	1.51	2.39	Auxin responsive GH3 gene family
FN01004.1.g	1.49	2.89	Auxin responsive GH3 gene family
FN01628.1.g	-4.22	-3.97	Auxin responsive GH3 gene family
FN03063.1.g	-6.17	-1.66	SAUR family protein
FN03133.1.g	1.16	1.56	BR-signaling kinase
FN03185.1.g	-1.61	-2.25	SAUR family protein
FN04245.1.g	1.96	2.65	Transcription factor MYC2
FN04959.1.g	-1.09	-1.75	Transcription factor TGA
FN08651.1.g	1.78	1.64	Auxin-responsive protein IAA
FN10311.1.g	-1.24	-1.36	Histidine kinase 3
FN12036.1.g	1.54	1.08	Cyclin D3
FN12857.1.g	1.4	2.06	Histidine kinase 4
FN15644.1.g	1.63	2.83	Auxin responsive GH3 gene family
FN18674.1.g	1.52	2.48	Histidine kinase 6
FN19201.1.g	1.93	2.35	Two-component response regulator ARR-A family
FN20151.1.g	-1.25	-1.01	Protein phosphatase 2C
FN27264.1.g	2.1	2.26	Auxin-responsive protein IAA
FN27301.1.g	1.43	2.52	Histidine kinase 4
FN31094.1.g	-5.14	-4.54	Auxin responsive GH3 gene family
FN33877.1.g	-1.03	-1.76	SAUR family protein

Supplementary Table S9. The sequence of gene-specific primers

Number	Gene ID	Forward primer sequence (5' to 3')	Reverse primer sequence (5' to 3')
1	Cannabis_sativa_newGene_11496	ACAACAATGAGACATACACCAAA	CCCAAACTGAGAAAGCACTAC
2	Cannabis_sativa_newGene_12033	GCAAGTGGTGGTGTTAGTGTT	GCTCCTCCTCCGACTCTACT
3	Cannabis_sativa_newGene_1215	AGGAGTTATCATTCTTCCAGACA	GTTCACCTTATCTTCATCCCA
4	Cannabis_sativa_newGene_12744	AAATGGGTAAGCAAGATAGCAC	CCTGAGCAAAGTAAATGGAAA
5	Cannabis_sativa_newGene_15493	GATGGAGCCTGAGTGTGATAA	CGTAGTTCTTGTTCATTGAGTCC
6	Cannabis_sativa_newGene_15537	CTATCTCTCCCTCTCACACACTC	TATAATGATCCACTCCTGCCT
7	Cannabis_sativa_newGene_16510	GTGAAACAAATTAAATACATGGAAA	TACACAAATGAAGCCAATATCTCT
8	Cannabis_sativa_newGene_16571	AAAGATCCCAACTACACGACA	CCTACCGATACAAGCAGAAATC
9	Cannabis_sativa_newGene_17026	ATCAACAGGACAGATTGGGTA	GAGAAATATCAAGCAAGTGCAA
10	Cannabis_sativa_newGene_18682	TATGCAGGAAGCAGTGGTAAT	GTAATGGCCTCAACAGAGAGA
11	Cannabis_sativa_newGene_20881	TTTGTATGGTCTTTGCTGGAT	TTTGGGTATCTCTTGAGCTTG
12	Cannabis_sativa_newGene_23402	CTTTCTTTCCCTGCTCTGTTT	AAGGTTGGTGTGTAATGATGTG
13	Cannabis_sativa_newGene_9451	CGGTTTCTCTATCAATCCTTTC	GGAGTTTCTTCTTTCATCTGCT
14	FN01628.1.g	AAGTCCGAAACAAAGACACC	AAGGAATCAGGGCAAAGAA
15	FN08139.1.g	ATGCTGCTGCTTATCATACCT	ACCTCCTTGTTCCTTCTGTTT
16	FN09070.1.g	GGAGAGGAGGAAGCCCTAT	CAGGACGATCAACCAACAT
17	FN22313.1.g	TTCTCCTTCACTCTTTCTCCTAA	CTAACTTTATGGCTGGTTTGTG
18	FN28192.1.g	GTACTTTGGCCTCATTTGTTG	CTCTGGCATTGTCATGTTGTA
19	FN31094.1.g	ACCATACGACCCTTACACCA	GCAGAGACCACAGAGGAGTT
20	FN33579.1.g	ATGGAAGCAAATACAGGGTCT	ATCGTAATGGTGGATGAGAAA
21	FN33773.1.g	CAACAAGGATTCAGAGGGTAA	ACGCACAAATAAACTTGGTAAA
22	GAPDH	AATGAGCACGAATACAAGTCAG	AAGAGAATGAAATGAAATGAACC

Supplementary Table S10. qRT-PCR validation of DEGs

Number	Gene ID	Relative gene expression by RNA-Seq		Relative gene expression by qRT-PCR	
		D2 vs. DS2	D4 vs. DS4	D2 vs. DS2	D4 vs. DS4
1	Cannabis_sativa_newGene_11496	-2.93	-6.72	-0.36	-3.41
2	Cannabis_sativa_newGene_12033	-5.32	-2.11	-1.20	-1.70
3	Cannabis_sativa_newGene_1215	-3.60	-3.21	-1.53	-2.82
4	Cannabis_sativa_newGene_12744	-3.42	-4.13	-1.22	-1.65
5	Cannabis_sativa_newGene_15493	-2.91	-3.71	-1.35	-1.46
6	Cannabis_sativa_newGene_15537	-2.76	-2.32	-1.06	-1.59
7	Cannabis_sativa_newGene_16510	-2.61	-2.24	-1.37	-1.71
8	Cannabis_sativa_newGene_16571	4.78	6.35	3.75	2.43
9	Cannabis_sativa_newGene_17026	-4.45	-4.73	-1.53	-1.07
10	Cannabis_sativa_newGene_18682	-3.01	-2.39	-1.27	-2.83
11	Cannabis_sativa_newGene_20881	-2.51	-2.38	-1.30	-1.24
12	Cannabis_sativa_newGene_23402	-3.58	-3.14	-1.36	-1.70
13	Cannabis_sativa_newGene_9451	1.47	2.24	0.75	0.62
14	FN01628.1.g	-4.22	-3.97	-1.87	-3.28
15	FN08139.1.g	-2.46	-3.01	-1.67	-1.33
16	FN09070.1.g	3.05	2.88	5.59	5.64
17	FN22313.1.g	-3.22	-2.69	-0.82	-3.03
18	FN28192.1.g	1.01	-1.13	1.69	-2.35
19	FN31094.1.g	-5.14	-4.54	-0.81	-2.88
20	FN33579.1.g	2.79	4.78	0.35	0.93
21	FN33773.1.g	-3.95	-4.00	-0.84	-2.99
